# Energy and Molecules from Photochemical/Photocatalytic Reactions. An Overview

**DOI:** 10.3390/molecules20011527

**Published:** 2015-01-16

**Authors:** Davide Ravelli, Stefano Protti, Angelo Albini

**Affiliations:** PhotoGreen Lab, Department of Chemistry, University of Pavia, via Taramelli 12, Pavia 27100, Italy; E-Mails: davide.ravelli@unipv.it (D.R.); prottistefano@gmail.com (S.P.)

**Keywords:** photochemistry, photocatalysis, energy storage, green chemistry

## Abstract

Photocatalytic reactions have been defined as those processes that require both a (not consumed) catalyst and light. A previous definition was whether such reactions brought a system towards or away from the (thermal) equilibrium. This consideration brings in the question whether a part of the photon energy is incorporated into the photochemical reaction products. Data are provided for representative organic reactions involving or not molecular catalysts and show that energy storage occurs only when a heavily strained structure is generated, and in that case only a minor part of photon energy is actually stored (*ΔG* up to 25 kcal·mol^−1^). The green role of photochemistry/photocatalysis is rather that of forming highly reactive intermediates under mild conditions.

## 1. Introduction: Photochemistry for Synthesis and Energy

More than a century ago, Giacomo Ciamician compared the way both Nature and chemistry practitioners produced complex molecules. Chemists had been extracting a variety of molecules from organisms and had demonstrated that, in most cases, the same structures could be built artificially. However, a key difference remained in the way such products were formed. In particular, plants appeared to be endowed by a “guarded secret” that made them able to synthesize complex structures under mild conditions, in strong contrast with the harsh conditions most often required for the generation of the same molecules in the laboratory. What could be the cause of such a difference? Ciamician thought this was the fact that they absorbed light. That is why he had embarked in a series of experiments and had found that solar light was actually able to cause a wealth of reactions. He was impressed by the smooth formation of new molecules under photochemical conditions. As an example, carbon-carbon bonds were formed upon irradiation and did not require a base, as it on the contrary occurred with the most typical C-C bond forming thermal reaction (known at that time), aldol condensation [1,2,3,4]. As a matter of fact, this issue was the subject of a dispute between Ciamician and the other “father” of organic photochemistry, Emanuele Paternò. Indeed, the latter scientist lamented that Ciamician had not grasped the potential of photochemistry in synthesis, and had limited himself to redox reactions of little synthetic significance [5]. Ciamician defended his ideas and evidenced the preparative significance of his work [6]. He also addressed the energetic aspect, with the consideration that fossil fuels are a sort of mineralized solar energy and are regenerated at a negligible rate with respect to the consumption by mankind, so that they are bound to finish in a few centuries. Therefore, according to Ciamician’s idea, mankind should have learnt to use directly solar light, thus having at its disposal not only a boundless resource, but also a clean environment, abandoning the dirty world based on burning fuels [5].

**Scheme 1:**
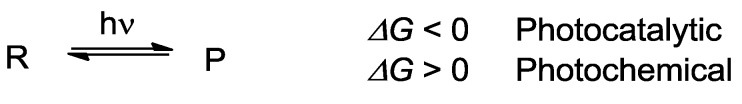
The effect of light on a chemical equilibrium and the historical definitions adopted.

From the energetic point of view, the key issue for classification was: in which way is the chemical equilibrium affected by light? A century ago, it was defined that the effect of light could be classed either as *photocatalytic* (photoaccelerating a process in the same direction in which it occurred spontaneously in the dark, *ΔG* < 0) or *photochemical* in a full meaning (proceeding counter thermodynamically by incorporating part of the energy of the absorbed quantum in the product, *ΔG* > 0; Scheme 1, where R: reagent, P: product) [7,8]:

The word photocatalytic reemerged much later with reference to a class of photochemical reactions, *viz*. to those processes where both light (that is consumed) and a catalyst (that is not) are required. Actually, the word photocatalysis designs a continuously developing area that since a couple of decades is the most extensively investigated subdiscipline of photochemistry.

The question is now, whether photocatalysis is a branch of the more general discipline of chemical catalysis. According to the IUPAC “Gold Book” [9], a catalyst is “*a substance that increases the rate of a reaction without modifying the overall standard Gibbs energy change*”. Moreover, “*a catalyst is both a reagent and a product of the reaction*” (Equation (1)). It is further added that “*the words catalyst and catalysis should not be used when the added substance reduces the rate of reaction*” and that “*the term catalysis is also often used when the substance is consumed in the reaction (for example: base-catalysed hydrolysis of esters). Strictly, such a substance should be called an activator.*” This must be distinguished from a chain process:

R + CAT → P + CAT(1)

R + hν → R* → P(2)

R + CAT + hν → R + CAT* → R* + CAT → P + CAT(3)

R + CAT + hν → R + CAT* → P + CAT(4)

In a light-induced reaction related definitions are used, but things are by necessity more complex. The chemical reaction involves either the reagent that has absorbed the photon (direct photochemistry, Equation (2)) or some not absorbing molecule (Equations (3) and (4)). In the latter case, if the light absorbing molecule is not consumed, it behaves like a catalyst and can be excited again. Two cases have to be distinguished, *sensitization* (Equation (3)) that involves a physical energy transfer between the absorbing molecule and the non-absorbing one (thus arriving again to an excited state of the reagent, though indirectly), and *photocatalysis* that involves any sort of chemical activation (such as the transfer of an electron or an atom; Equation (4)).

Sensitization is a simple and convenient way to carry out a photochemical reaction when the reactive excited state is not reached efficiently through the spectroscopic states (e.g., the triplet state of organic molecules or the singlet state of molecular oxygen). As for photocatalysis, according to the Gold Book [9] this is defined as a “*change in the rate of a chemical reaction or its initiation under the action of ultraviolet, visible or infrared radiation in the presence of a substance—the photocatalyst—that absorbs light and is involved in the chemical transformation of the reaction partners*”.

**Scheme 2:**
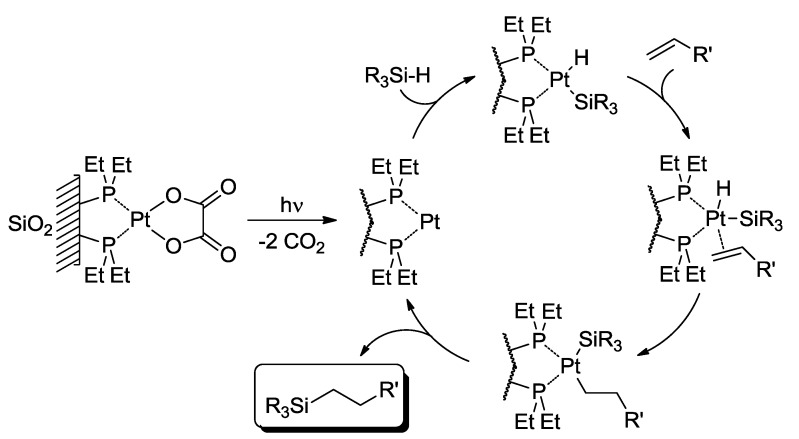
Hydrosilylation of olefins catalyzed by a (supported) Pt complex photochemically generated *in situ* [10].

From the point of view of the mechanism of photocatalytic reactions, it is convenient to distinguish three cases. In the first case (Equation (5)), a thermal catalyst is produced photochemically and promotes a thermal reaction. This is simply a variation of thermal catalysis, in which an inactive precursor CAT_I_ is activated photochemically. The quantum yield of formation of the product may be > 1. An example is reported in Scheme 2, where the photoinduced generation of a thermally active Pt complex allows the hydrosilylation of olefins [10]:

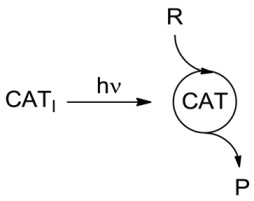
(5)

**Scheme 3:**
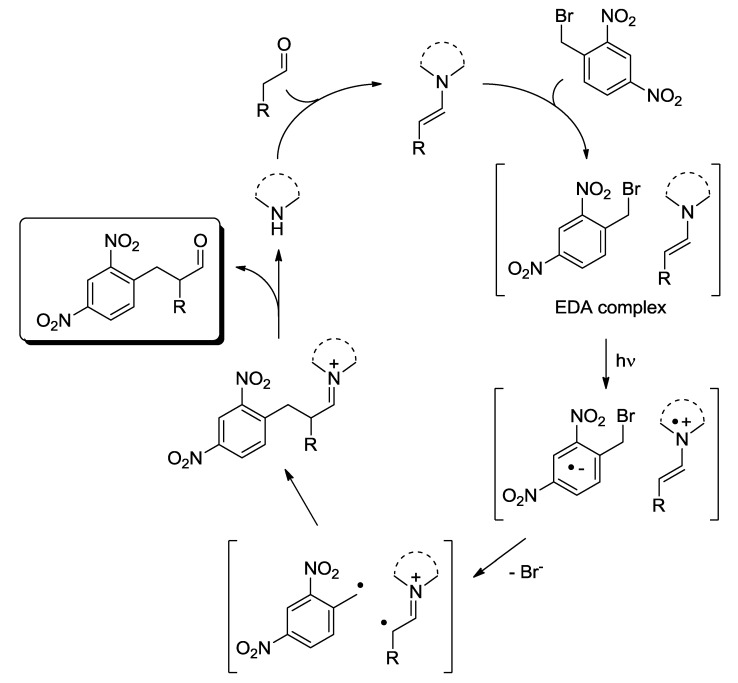
Organocatalytic (asymmetric) α-benzylation of aldehydes via an intermediate EDA (Electron Donor-Acceptor) complex under visible light irradiation [11].

In the second class, an unstable complex is formed between catalyst and reagent (R'---CAT', Equation (6)). This is a different, ground-state species with its own absorption band. Irradiation in such a band causes the reaction and regenerates the catalyst. Examples are found in many fields with various mechanisms of complexation, including the interaction between Lewis/Brønsted acids or bases, charge-transfer complexes (see the example reported in Scheme 3), reversible condensations as in the case of the transient formation of enamines in organocatalysis by interaction between a carbonyl function and an amine [11], but also adsorption on the surface of a solid material, whether participating in the photochemical reaction (typically a semiconductor) [12] or not [13,14]. The unifying idea is that the irradiated species is in some way modified before turning the light on, and at least in principle, it is possible to irradiate selectively the complexed/adsorbed species. The quantum yield of the overall process is < 1. Semiconductor photocatalysis, by far the largest topic in this field, is in fact usually discussed in terms of electron/hole transfer to the *adsorbed* molecule [12].


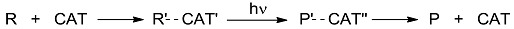
(6)

**Scheme 4:**
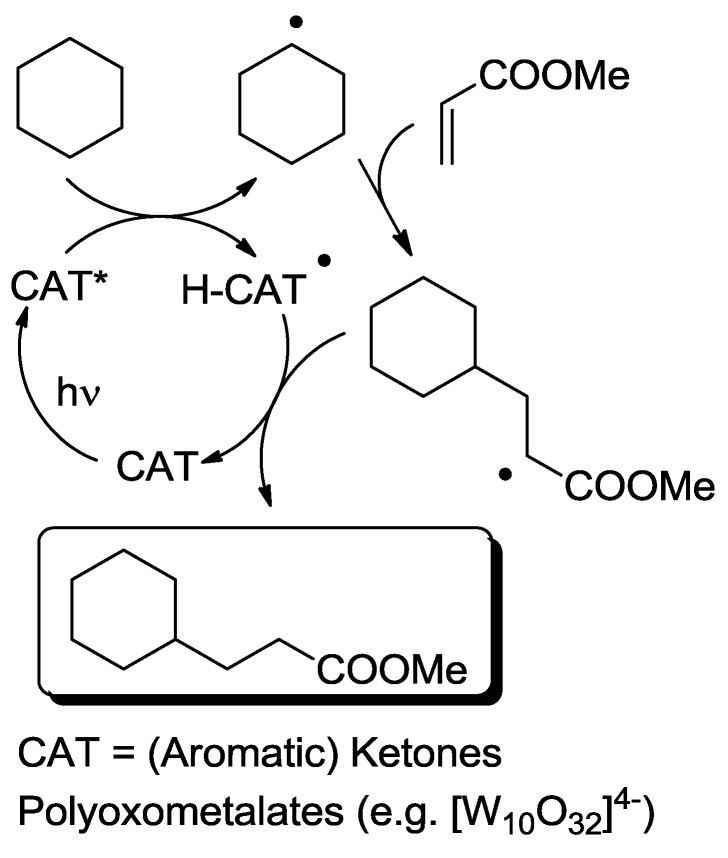
Photocatalyzed conjugated radical addition to electron-poor olefins promoted by excited ketones and polyoxometalates [15].

**Scheme 5:**
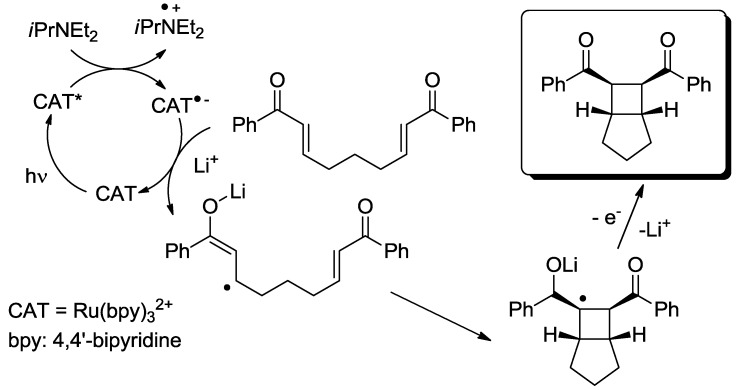
Photocatalyzed cyclobutane ring formation exploiting a tertiary amine as a sacrificial electron donor [17].

In the third class, a catalyst is excited and interacts with a ground state reagent. Some chemical process causes the reaction of the reagent that is transformed into another species that gives the final product, thereby regenerating the catalyst in the original form (Equation (7)). A typical example (see Scheme 4) is the Hydrogen Atom Transfer (HAT) process from suitable H-donors (e.g., alkanes) promoted by excited ketones and polyoxometalates to give a radical species, then exploited in C-C bond forming reactions [15]. An alternative approach involves the interaction with some reagent Z (e.g., a sacrificial electron donor or acceptor) [16] that brings the photocatalyst into an “active” form CAT' then causing the reaction (Equation (8)). In a typical example, the excited state of a transition metal complex is reduced by a suitable electron donor (e.g., a tertiary amine) to give the “active” form that actually converts the reagent. In some way, the process then regenerates the photocatalyst (see the example reported in Scheme 5) [17]. The quantum yield is again < 1. A synthetically quite promising method involves the merging between organocatalysis and photocatalysis, particularly when activated by visible light. This path is enjoying an explosive development based on joining the experience on transition metal complexes as electron transfer sensitizers and organocatalysis, with the formation in equilibrium of active intermediates [18,19,20,21,22,23].


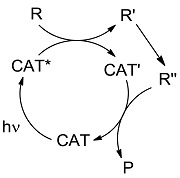
(7)


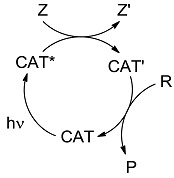
(8)

After that attention in this field has been long concentrated on the degradation of pollutants and on the conversion of solar radiation into a convenient energy vector, as in semiconductor photocatalytic water splitting yielding hydrogen, synthetic approaches exploiting the methods above have now been conquering more attention. In this sense, the reaction of third class are simpler and easier to explore by physicochemical methods. However, those of the second class are more promising, because the photochemical reaction involves a prearranged, ground state complex, rather than having to count on the reaction of the short-lived excited states. The long contact time gives further possibilities of governing the reaction and its selectivity. These are the best conditions for “green” chemistry.

Apart from the first class, which is clearly something different, which is the fundamental difference between catalysis and photocatalysis? Some years ago, Ohtani observed that “*the most significant difference between photocatalysis and catalysis lies in their thermodynamics*” [24]. In a general definition, a catalyst reduces the activation energy of a given chemical reaction by changing the intermediate states and thereby accelerates the reaction which proceeds spontaneously with negative Gibbs energy change, that is, catalysis is limited to thermodynamically possible reactions. On the other hand, it is well known that photocatalysis can drive energy-storing reactions, for examples splitting water into hydrogen and oxygen. In this sense, “photocatalysis” must be recognized as a concept completely different from that of “catalysis”.

**Scheme 6:**
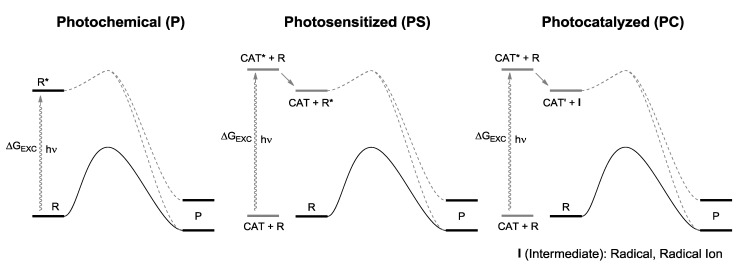
Definition of the different light-induced processes examined in Table 1.

If this criterion is important, then one should assess for every light-induced process, whether it occurs by direct irradiation (P), photosensitization (PS), or photocatalysis (PC), the position of the thermal equilibrium and the effect of a photon on it (see Scheme 1 above). With few exceptions, these data seem not to be available at present [25]. In order to explore in which way light induced processes occur from the energetic point of view, whether storing or consuming energy, we carried out a series of calculations through a uniform approach for typical photochemical, photosensitized and photocatalytic reactions (see Scheme 6 for the definition of the data reported) at the G3(MP2)B3 level (see Supplementary Materials for the computational details).

## 2. Results and Discussion

The obtained results are gathered in Table 1, where the Gibbs free energy changes (*ΔG*) between ground states of reagents and products, along with the energy of the activating radiation (*ΔG*_EXC_), are listed. The energy of the activating light is meant as the minimum required, that is the less energetic radiation that is absorbed and makes the reaction run. As for photocatalytic reactions, processes involving a molecular photocatalyst soluble in the reaction medium have been chosen rather than those where the reaction occurred on the surface of a solid semiconductor material [26,27]. As a consequence, the reactions can be safely compared with each other since in all of them only molecular species in solution are involved, independently from the class they belong to, avoiding to have to treat adsorption phenomena. A perusal at the Table evidences different groups with reference to the type of process and the thermodynamic of the overall reaction.

*Oxidations.* These are strongly exergonic reactions, whichever is the mechanism, via electron transfer or singlet oxygen (Table 1, entries 1, 2) [28,29,30]. The irradiation wavelength used is that absorbed by the sensitizing dye.

**Table 1:** Calculated thermodynamic parameters at the G3(MP2)B3 level of theory for exemplificative photochemical reactions. These are classified as occurring either via direct irradiation (P), or under photosensitized (PS) or photocatalytic (PC) conditions (see Scheme 6).

Entry	Reaction	Class of Reaction	*ΔG*_EXC_^a^	*ΔG* ^a^
			(λ_EXC_) ^b^	( *ΔH*) ^a^
**1 [28]**	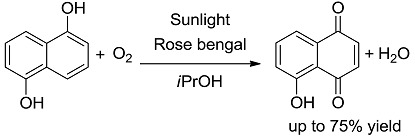	PS/PC	53 (540)	−80.24 (−80.74)
**2 [30]**	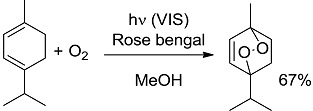	PS	53 (540)	−11.52 (−25.16)
**3 [31]**	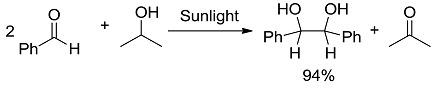	P	94 (304)	−3.05 (−13.99)
**4 [32]**	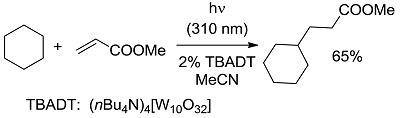	PC	73 (392)	−11.22 (−22.05)
**5 [15,33]**	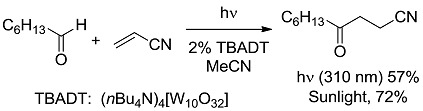	PC	73 (392)	−17.00 (−28.89)
**6 [34]**	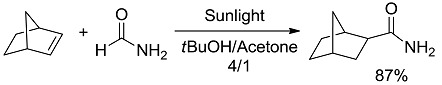	PS	92 (311)	−18.34 (−31.13)
**7 [35]**	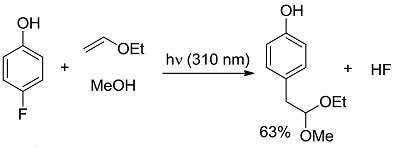	P	95 (301)	−19.31 (−32.89)
**8 [36]**	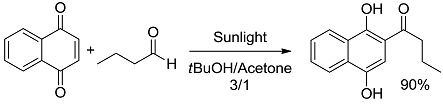	P	77 (371)	−18.55 (−32.75)
**9 [37]**	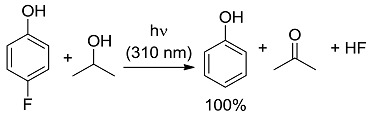	P	95 (301)	−17.91 (−6.15)
**10 [38]**	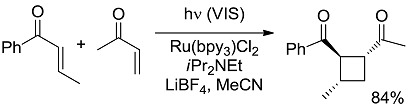	PC ^c^	58 (493)	−6.91 (−20.86)
**11 [39]**	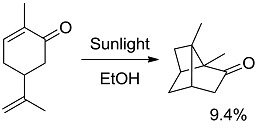	P	119 (240)	3.74 (0.27)
**12 [40]**	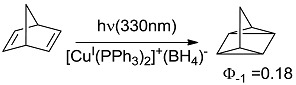	PS	87 (330)	24.63 (24.28)
**13 [41]**	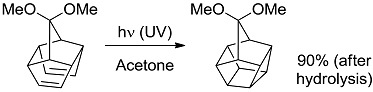	PS	92 (311)	19.86 (18.43)
**14 [42]**	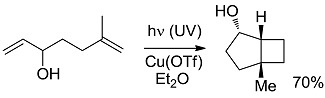	PC	89 (321)	−8.72 (−12.90)
**15 [43]**	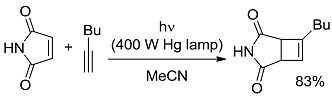	P	−	−17.92 (−31.43)
**16 [44]**	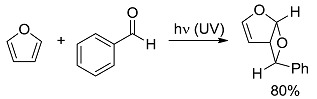	P	94 (304)	20.75 (7.53)
**17 [45]**	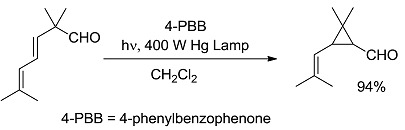	PS	84 (340)	8.71 (8.16)
**18 [46]**	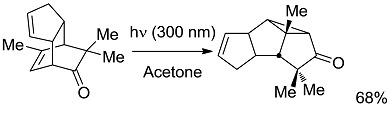	PS	92 (311)	9.60 (9.57)
**19 [47]**	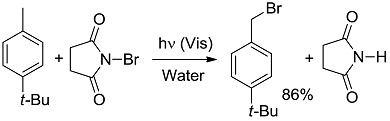	P	−	−24.25 (−24.71)
**20**	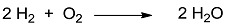	-	−	−107.2 (−113.5)
**21**	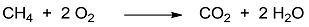	-	−	−193.6 (−193.9)
**22 [48]**	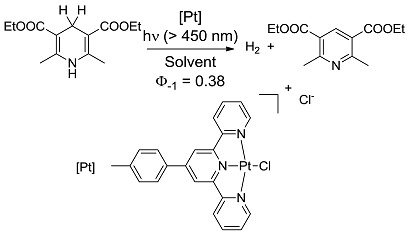	PC	67 (427)	−5.77 (1.40)

^a^ Values expressed in kcal·mol^−1^; ^b^ Values expressed in nm; ^c^ In the examined reaction, compounds *i*Pr_2_NEt and LiBF_4_ act as sacrificial reductant and Lewis acid, respectively.

*Carbon-Carbon (or C-H) bond forming reactions*. When occurring via coupling of stabilized radicals, such reactions are weakly exergonic, as shown in the case of the pinacolic dimerization (entry 3) [31]. Reactions involving addition across a C=C double bond are the most exergonic, either when a carbon based radical (compare alkyl [32], acyl [33], and carbamoyl radicals [34], entries 4–6) or a photogenerated carbocation [35] (compare the phenyl cation-based arylation of nucleophilic alkenes, see entry 7) are involved. C-C bond formation is strongly exergonic also when acylation of a quinone ring is accompanied by conversion to hydroquinone (entry 8) [36]. Reduction of an aggressive, oxidant intermediate, such as a phenyl cation, is likewise strongly exergonic (entry 9) [37].

*Small ring-forming reactions.* Three and four-membered rings are notorious for the high energy connected to their strained structures. Thus, formation of cyclobutanes is exothermic when starting from two conjugated ketones and conserves both C=O groups (entry 10) [38], but slightly endothermic when only one of the C=C bonds is conjugated (entry 11) [39] and markedly so when both are isolated (entries 12, 13) [40,41], although the reaction turns exergonic when the molecule is not prearranged in a convenient conformation (entry 14) [42]. Notice, however, that cyclobutene formation from an alkyne is exergonic (entry 15) [43]. Oxetane formation is strongly endergonic (entry 16) [44]. Three-membered ring-formation likewise brings in endothermicity, as in the case of di-π-methane (entry 17) [45] and oxa-di-π-methane (entry 18) [46] rearrangements.

*Other reactions*. Formation of a compound containing a bond between carbon and an electron-withdrawing atom are strongly exergonic (entry 19) [47], as are of course combustions, included for the sake of comparison with high-energy thermal reactions (entries 20, 21). Notice that when the two last reactions are considered in the contrary direction, one has water-splitting or CO_2_ reduction to methane (a 8-electron process), that are much more strongly endergonic than any of the above organic reactions and are typically studied by heterogeneous photocatalysis. In contrast, the almost thermoneutral oxidation of Hantzsch dihydropyridine (entry 22) [48] may be taken as a simplified example of processes occurring in living cells, where energy and “reducing power” are exchanged through small steps. It is apparent that at most a small fraction of the overall energy furnished to the system (*ΔG*_EXC_) is stored as chemical energy in the products. Thus, in the most studied case, that of the rearrangement of norbornadiene (entry 12), most of the energy impinging is directly converted to heat during the conversion to quadricyclane.

**Figure 1:**
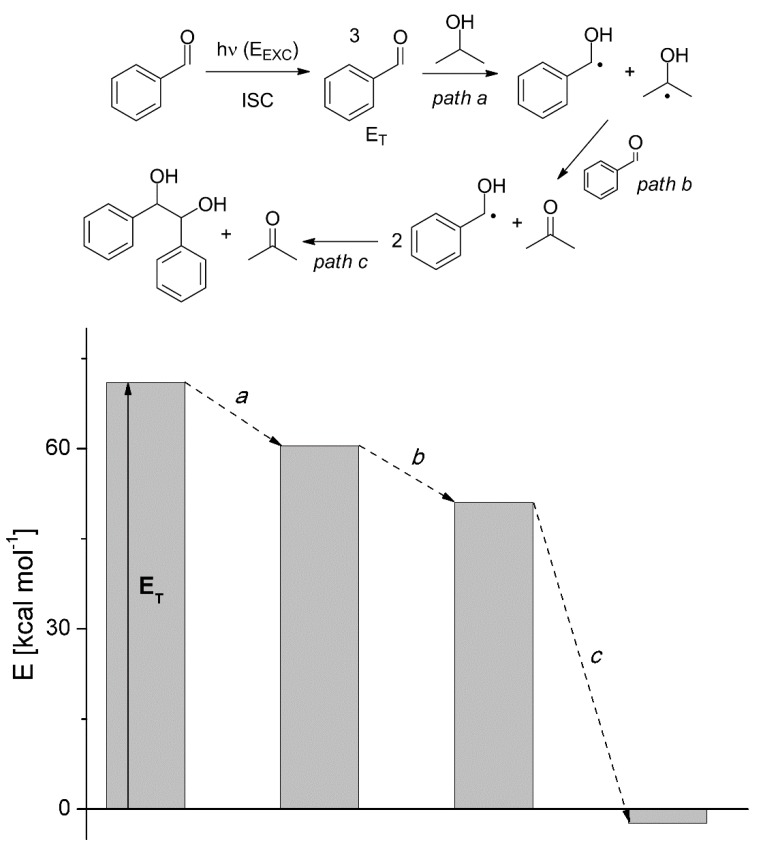
The energetic profile of the steps involved in the reaction reported in Table 1, entry 3. Adapted from Ref. [49].

*Thermodynamics*. As it is well known, comparing photochemical and thermal reactions is inappropriate, because excited states are different species, with a thermodynamic behavior of their own, not related to that of the ground states. Overall, photochemical reactions occur through different Potential Energy Surfaces (PESs). Molecules are promoted to the excited state with no geometric change and from there chemistry begins, until at some point they return to the ground state on a different PES and at a different atom configuration, that is to the product. The final product energy may lie either below or above the starting state of the reagent. It may be useful wondering at which point the system drops down. This may be illustrated by monitoring the bimolecular reduction of an aromatic carbonyl, an almost thermoneutral reaction. As it appears from Figure 1, formation of the intermediates, C-centered radicals, from triplet benzaldehyde and isopropanol involves only a moderate energy drop (steps *a*, *b*), while most of the decrease occurs at the bond formation stage (step *c*). In other words, most of the light energy absorbed is used in the bond breaking reaction, and then the high energy intermediates lose their energy by forming new bonds.

**Figure 2:**
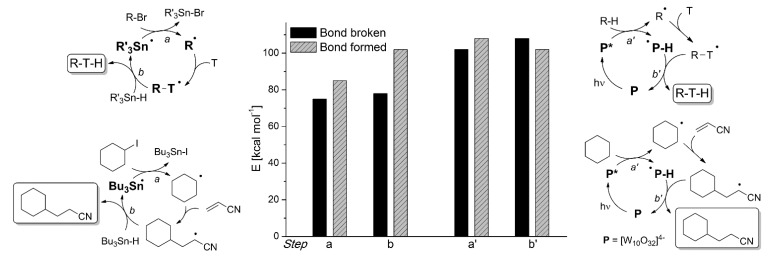
Chain *vs.* non-chain, photocatalytic process. The energy change associated to each step is considered. In the thermal chain process [50], both steps *a* and *b* involve the formation of a bond that is more energetic than the one that is cleaved, and the overall process involves relatively low energies. On the contrary, steps *a'* and *b'* in the photocatalytic process [15] involve the cleavage of a strong bond and the endergonic nature of the latter step precludes a chain process. Adapted from Ref. [49].

*Photocatalysis.* The activation by the photocatalyst occurs by either of the two processes, atom transfer and electron transfer. A typical C-C bond forming reaction would be the addition of an organic compound R-H across a π-bond (see compound T, standing for “trap” in Figure 2; a well known example is an electron-poor olefin, such as acrylonitrile). The overall process is largely exothermic (see the related case reported in entry 4, Table 1), but requires the generation of an alkyl radical and thus the cleavage of a C-H bond, a process involving too high an energy for being amenable to laboratory conditions. The common way out is inserting a radicofugal group, as in the well known Giese method from alkyl halides via stannyl radicals as chain carriers [50]. The alkyl radicals are generated by transfer of a halogen atom X (Br, I) to a R_3_Sn^•^ radical (path *a* in Figure 2) and the radical adduct abstracts a hydrogen atom from a trialkylstannane R_3_SnH (path *b*). The two processes are exergonic, *viz*. it is more convenient to make a Sn-X than a C-X bond, and a C-H than a Sn-H bond. In other words, the bond formed is always stronger than that broken (accordingly, black bars are smaller than grey bars for steps *a* and *b* in Figure 2). Thus, every cycle is exergonic and the process goes on. Photocatalysis has however more possibilities [51]. In fact, hydrogen transfer to an excited state that has a radical character is viable, in particular with triplet carbonyls, polyoxometalates and related species, where the excited state is similar to an alkoxy radical and thus hydrogen transfer forms a strong O-H bond (step *a'* in Figure 2). In the typical embodiment, alkylation by the radical formed by hydrogen abstraction follows. Thus, a catalytic, non-chain process is possible, with a quantum yield < 1, but occurring under very mild conditions.

## 3. Experimental Section

The computational investigation has been carried out in order to recognize the energy change associated to the reaction under consideration. All of the simulations have been carried out with the Gaussian09 Software [52]. See Supplementary Materials for further details.

## 4. Conclusions

Both “catalytic” and “non-catalytic” photochemical reactions offer a wide-scope potential for a contribution to sustainability, that is green chemistry, by generating high-energy intermediates with the least possible exertion, coming down from above, from the high-lying excited state, not confronting a difficult ascending path from the ground state (as it occurs in thermal processes). These reactions appear to be a topic of great promise, although expensive from the energetic point of view. However, the mild conditions of the photochemical approach offer an unparalleled advantage and often allow to start from little-functionalized reagents via shorter paths [53,54]. As long as solar/visible light irradiation becomes used (a few examples are reported in Table 1), the potential of photocatalysis for organic synthesis should be fully developed [18,19,20,21,22,23,27].

As for energy storage, it is difficult to build molecules of very high energy content. The covalent bond is synonym of stability and even in the more favorable cases, in molecules specifically built for this aim, only a fraction of the energy of the photon (often around 10%–15%; as much as 25% in the most favorable instances) can be stored as chemical energy. If then one takes into account that exploiting such an energy requires overcoming a further barrier, and thus wasting part of the gain, it can be concluded that organic reactions (characterized by a small positive *ΔG*) are unlikely to become the main contribution of photochemistry to a sustainable world. The real energetic challenge remains limited to simple systems, such as water splitting and CO_2_ reduction, where molecules not absorbing directly solar light are involved. Thus, these systems require a photocatalyst, allowing for the energy from an appropriate number of photons to be transferred into a molecule of a convenient energy vector, such as H_2_ or CH_4_ [55].

## Supplementary Materials

Supplementary materials can be accessed at: http://www.mdpi.com/1420-3049/20/01/1527/s1.
