# Supplementary material for: Energy and Molecules from Photochemical/Photocatalytic Reactions. An Overview
Source: Molecules. 2015 Jan 16;20(1):1527–42. doi: 10.3390/molecules20011527 (PMC6272209; doi:10.3390/molecules20011527)
Supplement: Supplementary file 1 [file molecules-20-01527-s001.pdf]

Supplementary Materials

Computational Details

The computational investigation has been carried out in order to recognize the energy change associated to the reaction under consideration. All of the simulations have been carried out with the Gaussian09 Software [1].

Preliminary calculations have been performed using Density Functional Theory (DFT) in order to find out the most stable conformer of the molecule under investigation. Thus, reactants and products have been initially optimized adopting the B3LYP [2–5] functional and the standard 6-31G(d) basis set in the gas phase. To confirm the nature of the stationary points, vibrational frequencies (in the harmonic approximation) were calculated for all of the optimized structures at the same level of theory as geometry optimizations and it was verified that local minima had only real frequencies. In several cases, the reactions had well defined stereochemical outcomes and the corresponding products have been modeled. However, when the stereochemistry of the reagents/products was not dealt with in the original reference or mixture of stereoisomers could be involved, only the most stable isomer was considered for the computational investigation.

For each molecule, the most stable conformer has been used as the starting point for a refinement by adopting a higher level of theory, *viz.* using the composite method G3(MP2)B3 [6] in the gas phase. This approach is particularly advantageous for medium-large systems (as those considered here) and offers a very good performance while limiting the computational cost. This method is a variant of the G3(MP2) theory based on geometries and zero point vibrational energies calculated at the same level as above, that is B3LYP/6-31G(d). The steps required for the calculation of the desired energy values include:

- Optimization at the B3LYP/6-31G(d) level of theory
- Frequency calculation at the B3LYP /6-31G(d) level of theory
- Single point calculation at the QCISD(T,FC)/6-31G(d)//B3LYP/6-31G(d) level;
- Single point calculation at the MP2(FC)/G3MP2large//B3LYP/6-31G(d) level.

The computed enthalpy and Gibbs free energy were used as obtained from the output in the gas phase, without introducing any correction, since the main objective of this work was the evaluation of the energy change associated with the reaction considered and was not intended to be directly compared with any experimental data.

Thermochemical data have been calculated adopting the default options, *viz.* temperature: 298.150 K and pressure: 1.00000 atm. Optimized geometry listed in cartesian format (coordinates are given in Å) are reported below. The conversion factor between Hartree and kcal·mol^{-1} has been: 1 Hartree = 627.509 kcal·mol^{-1}.

When summing the data for calculating the energy changes for the reactions considered, all the digits available from the calculations were used; nevertheless, the energy values reported below (in Hartree units) have been rounded considering 6 significant digits after the unit.

Table S1. Computed enthalpy and Gibbs free energy for the compounds describe in the text. These values have been used for calculating the reaction energy changes reported in Table 1 in the main text.

Compound	G3(MP2)B3 Enthalpy (H, Hartree)	G3(MP2)B3 Gibbs Free Energy (G, Hartree)
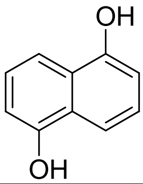	−535.526679	−535.571348
O_2	−150.168161	−150.191448
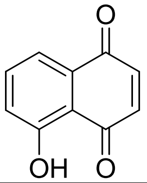	−609.481647	−609.527361
H_2O	−76.341863	−76.363309
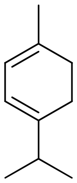	−389.945639	−389.994134
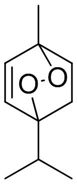	−540.153894	−540.203936
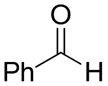	−345.027564	−345.065666
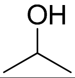	−194.034539	−194.068509
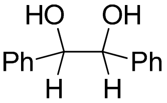	−691.264563	−691.322094
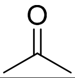	−192.847397	−192.882602
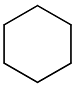	−235.407845	−235.443633
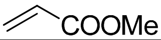	−306.027908	−306.066111

Table S1. *Cont.*

Compound	G3(MP2)B3 Enthalpy (H, Hartree)	G3(MP2)B3 Gibbs Free Energy (G, Hartree)
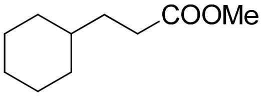	−541.470896	−541.527630
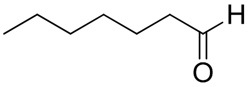	−349.775265	−349.823824
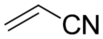	−170.567318	−170.598428
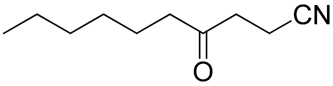	−520.388628	−520.449341
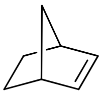	−272.228308	−272.263296
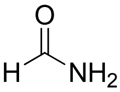	−169.669801	−169.700128
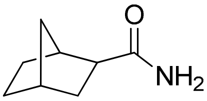	−441.947714	−441.992647
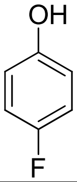	−406.148222	−406.186192
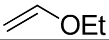	−232.046577	−232.082877
MeOH	−115.553223	−115.580233
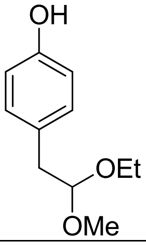	−653.441734	−653.501638
HF	−100.358704	−100.378439
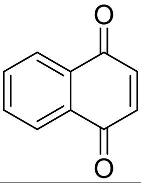	−534.320517	−534.364647

Table S1. *Cont.*

Compound	G3(MP2)B3 Enthalpy (H, Hartree)	G3(MP2)B3 Gibbs Free Energy (G, Hartree)
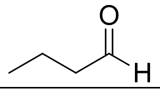	−232.070218	−232.107748
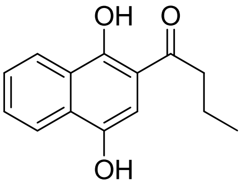	−766.442918	−766.501964
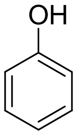	−306.986463	−307.022195
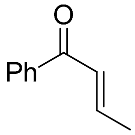	−461.533034	−461.581384
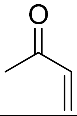	−230.869239	−230.905235
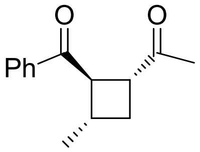	−692.435518	−692.497627
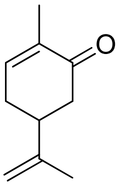	−463.904434	−463.955026
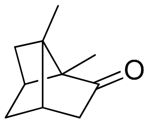	−463.903996	−463.949070
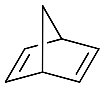	−271.003234	−271.036807
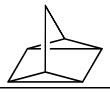	−270.964538	−270.997559
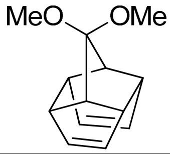	−654.336069	−654.387413

Table S1. *Cont.*

Compound	G3(MP2)B3 Enthalpy (H, Hartree)	G3(MP2)B3 Gibbs Free Energy (G, Hartree)
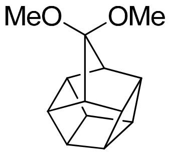	−654.306700	−654.355761
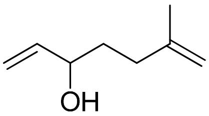	−387.788845	−387.838623
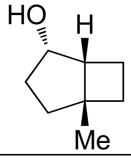	−387.809395	−387.852526
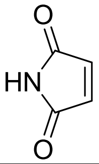	−358.957308	−358.993243
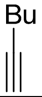	−234.149617	−234.190018
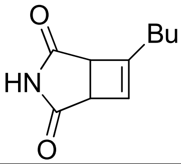	−593.157018	−593.211825
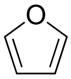	−229.674812	−229.705894
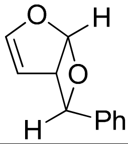	−574.690384	−574.738495
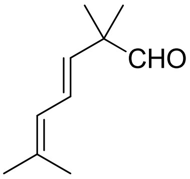	−465.080242	−465.136027
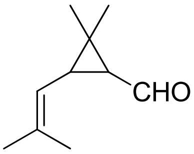	−465.067245	−465.122146

Table S1. *Cont.*

Compound	G3(MP2)B3 Enthalpy (H, Hartree)	G3(MP2)B3 Gibbs Free Energy (G, Hartree)
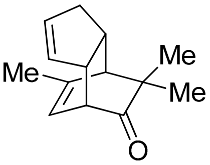	−618.477659	−618.531424
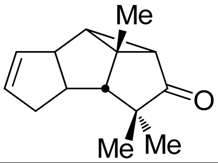	−618.462402	−618.516130
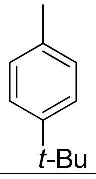	−428.023483	−428.074203
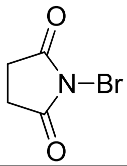	−2932.369583	−2932.412303
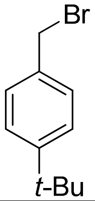	−3000.259962	−3000.315197
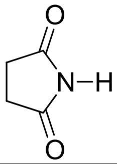	−360.172485	−360.209955
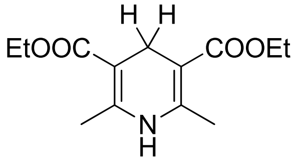	−861.188818	−861.262892
H_2	−1.167339	−1.182131
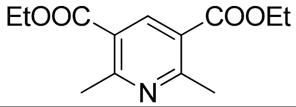	−860.019243	−860.089963
CH_4	−40.420548	−40.441690
CO_2	−188.382178	−188.406529

Cartesian coordinates

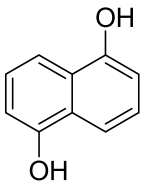

C	2.36324500	-0.91081600	-0.00000100
C	1.83538300	0.36406600	-0.00000900
C	0.42364300	0.57677100	0.00000900
C	-0.42364300	-0.57677100	0.00001100
C	0.13948800	-1.87984900	0.00001600
C	1.50704200	-2.03478200	0.00002700
H	0.51992300	2.73967800	0.00002500
H	3.44330000	-1.04905500	0.00000800
C	-0.13948800	1.87984900	0.00001300
C	-1.83538300	-0.36406600	-0.00000300
H	-0.51992300	-2.73967800	0.00005700
H	1.94282500	-3.03004800	0.00004300
C	-2.36324500	0.91081600	0.00001300
C	-1.50704200	2.03478200	0.00002400
H	-3.44330000	1.04905500	0.00001000
H	-1.94282500	3.03004800	0.00002300
O	-2.61601400	-1.48891300	-0.00004700
H	-3.54761200	-1.21953300	-0.00005800
O	2.61601500	1.48891300	-0.00003000
H	3.54761200	1.21953200	-0.00009100

O_2

O	0.00000000	0.00000000	0.60729600
O	0.00000000	0.00000000	-0.60729600

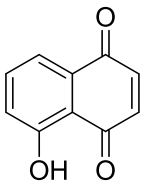

C	-2.27690500	-1.25344100	-0.00002400
C	-1.78166900	0.06546500	-0.00002100
C	-0.38217000	0.28338100	-0.00000200
C	0.49295700	-0.83218000	-0.00000300
C	-0.01129100	-2.12616200	-0.00000900
C	-1.40025500	-2.32711300	-0.00002300
H	-3.35283800	-1.39508900	-0.00001300
C	0.14843500	1.64856500	0.00002800
C	1.97059900	-0.62198000	0.00000900
H	0.68285800	-2.95934100	-0.00000300
H	-1.79610400	-3.33893600	-0.00002700
C	2.45855100	0.78102900	0.00000500
C	1.61685400	1.82805300	-0.00001700
H	3.53823000	0.90156000	0.00001200
H	1.96203200	2.85820100	-0.00006400

O	-2.66544600	1.07095600	0.00006000
H	-2.14272200	1.91522700	0.00018400
O	-0.58955100	2.65048900	-0.00005500
O	2.76723500	-1.55336000	0.00002800

H_2O

O	0.00000000	0.00000000	0.11972000
H	0.00000000	0.76156000	-0.47887900
H	0.00000000	-0.76156000	-0.47887900

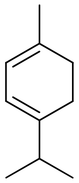

C	0.03942900	-1.03776700	-0.40872000
C	-1.36926000	-1.21233700	0.17937400
C	-1.60854400	1.22902700	0.02018200
C	-0.15508600	1.34730400	0.14324500
H	-0.02050100	-1.08775400	-1.50899900
H	-1.85726600	-2.08079500	-0.28111200
H	-2.19521000	2.14436100	-0.03904500
H	0.25993100	2.33062300	0.36003600
H	-1.29545300	-1.44326800	1.25642900
H	0.67129100	-1.88033200	-0.10427400
C	-3.71385100	-0.13856800	-0.04939600
H	-4.23087500	0.82563600	-0.09492400
H	-4.08934900	-0.68396300	0.82931400
H	-4.01385500	-0.72773800	-0.92780600
C	2.17386400	0.41249400	0.07929700
H	2.40175700	1.47527100	0.24135300
C	2.87865600	-0.02178000	-1.22120500
C	2.73447000	-0.36530100	1.28796200
H	2.55787200	-1.44336300	1.19068400
H	3.81770200	-0.21618200	1.37375600
H	2.26855000	-0.03106500	2.22126100
H	2.70705400	-1.08359100	-1.43516200
H	2.51849100	0.55705000	-2.07901400
H	3.96208800	0.12666200	-1.14081400
C	0.66662100	0.29246200	-0.02392100
C	-2.22333600	0.03087400	0.01623400

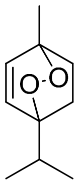

C	0.03248300	1.38574200	-0.72668300
C	-1.50483700	1.32348600	-0.52262400
C	-1.35293200	-1.12628300	-0.88046300
C	-0.03262600	-1.07199800	-1.06571200
H	0.45855900	2.18533400	-0.11610600

H	-1.83303500	2.09100400	0.18650200
H	-2.01410300	-1.86763600	-1.32210600
H	0.53073400	-1.76115500	-1.68854000
H	-2.04516300	1.48450300	-1.46213400
H	0.29475200	1.58681300	-1.77164700
C	-3.33365500	-0.20236900	0.44167300
H	-3.51992000	-1.18374400	0.88893900
H	-3.96288300	-0.09777300	-0.44905300
H	-3.61949400	0.56893300	1.16303600
C	2.19092900	-0.03088700	-0.33484000
H	2.44063200	0.00707400	-1.40632900
C	2.72484700	-1.35776600	0.23525600
C	2.88030900	1.15627800	0.35830700
H	2.56809000	1.23198600	1.40491100
H	3.96722400	1.01870600	0.33857800
H	2.66325400	2.10996300	-0.13282700
H	2.52229200	-1.42743500	1.30793100
H	2.26843700	-2.23002300	-0.24384600
H	3.80834300	-1.42232700	0.08433000
C	0.64500700	0.03484400	-0.28509000
C	-1.86800500	-0.05990900	0.05661700
O	0.30524500	-0.13712400	1.12190500
O	-1.15734900	-0.18800700	1.32306000

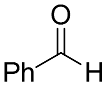

C	1.73576200	1.06060400	-0.00003100
C	0.36107600	1.29223900	0.00000600
C	-0.53398200	0.21467700	0.00003300
C	-0.04523300	-1.10089500	0.00004600
C	1.32640000	-1.33125700	0.00001900
C	2.21687300	-0.25091500	-0.00003300
H	2.42989800	1.89631900	-0.00006700
H	-0.02484800	2.30980200	-0.00001300
H	-0.75849900	-1.91958700	0.00007300
H	1.70768800	-2.34873800	0.00002600
H	3.28827100	-0.43341400	-0.00004800
C	-1.99240000	0.46891800	0.00005800
H	-2.27462700	1.54573900	0.00023700
O	-2.84735700	-0.39629400	-0.00010000

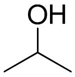

O	-0.03688200	1.37008600	-0.16628300
H	-0.87508800	1.77206400	0.11041700
C	0.00174900	0.04198600	0.36500800
H	-0.00621700	0.08441400	1.46807000
C	-1.20484100	-0.77448700	-0.10340700
C	1.32649600	-0.55987700	-0.08862400
H	-2.14518000	-0.31293000	0.22583800
H	-1.22008100	-0.83443600	-1.19754100
H	-1.17725900	-1.79285100	0.30190900
H	1.35941100	-0.62106200	-1.18232000

H	2.16105600	0.06528700	0.24389700
H	1.45798900	-1.56690700	0.32212600

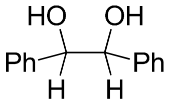

C	-0.42423000	-0.03774500	0.70336200
C	0.42392800	0.00885600	-0.60586300
H	-0.11438300	0.78974100	1.35282700
H	0.10652900	0.87943900	-1.19765800
C	-1.91125100	0.11188000	0.38113000
C	-2.79057200	-0.96944200	0.50347300
C	-2.41365900	1.34002900	-0.07474300
C	-4.14037900	-0.83036100	0.16856200
H	-2.41375700	-1.90883000	0.89489200
C	-3.76016000	1.48191600	-0.40629400
H	-1.74793100	2.19707600	-0.16100200
C	-4.62861700	0.39312000	-0.28933400
H	-4.81106500	-1.67924700	0.27425200
H	-4.13339400	2.44253400	-0.75169900
H	-5.67878200	0.50240800	-0.54634000
C	1.91119100	0.11583600	-0.33464800
C	2.77530800	-0.96918700	-0.51009700
C	2.43595200	1.33336900	0.11773400
C	4.13733500	-0.83918200	-0.23005100
H	2.38174700	-1.90883200	-0.88138200
C	3.79467200	1.46350800	0.40003500
H	1.77737000	2.19045100	0.24661500
C	4.65128900	0.37383000	0.22801600
H	4.79773600	-1.69062200	-0.37296400
H	4.18492200	2.41602100	0.74881300
H	5.71155000	0.47271600	0.44489300
O	0.17083100	-1.19665900	-1.33269200
H	-0.77489700	-1.19579300	-1.55894200
O	-0.13365400	-1.23703100	1.39504100
H	0.05209500	-1.89610200	0.70121800

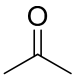

C	0.00000000	0.18530800	0.00000000
C	-1.29310100	-0.61479100	0.00000400
C	1.29309900	-0.61479400	-0.00000400
H	-2.14853100	0.06356300	0.00021200
H	-1.34153000	-1.26714800	-0.88109400
H	-1.34134300	-1.26749300	0.88085500
H	2.14853000	0.06355700	-0.00021500
H	1.34152700	-1.26714800	0.88109600
H	1.34133800	-1.26750000	-0.88085300
O	0.00000300	1.40097900	0.00000000

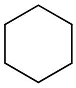

C	1.45211700	-0.21100900	0.22934500
C	0.54319900	-1.36271900	-0.22937300
C	-0.90873000	-1.15170600	0.22942400
C	-1.45211300	0.21100300	-0.22935400
C	-0.54320600	1.36271700	0.22936900
C	0.90873200	1.15171200	-0.22941100
H	-1.54777400	-1.96154300	-0.14589500
H	0.56845100	-1.42609700	-1.32770700
H	0.92467400	-2.32106600	0.14627100
H	1.51970200	-0.22063000	1.32764700
H	2.47292400	-0.35933000	-0.14630900
H	-1.51968000	0.22061200	-1.32765700
H	-2.47292800	0.35932800	0.14627600
H	-0.92467700	2.32106300	-0.14628700
H	-0.56847200	1.42610200	1.32770200
H	0.95049700	1.20468600	-1.32775100
H	1.54776800	1.96153900	0.14594300
H	-0.95047800	-1.20465500	1.32776700

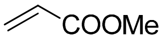

C	-0.04359800	0.11760100	-0.00005700
O	0.06062500	1.32782300	-0.00004800
C	-2.49176300	-0.01310400	0.00007700
H	-2.53026100	1.07257100	0.00023700
H	-3.43292400	-0.55451400	0.00009100
C	-1.31702900	-0.64641300	-0.00009500
H	-1.23949400	-1.72960900	-0.00019300
O	1.01687200	-0.72524800	0.00008400
C	2.30319300	-0.08855000	-0.00000900
H	2.42417200	0.53384100	0.89092300
H	3.03226900	-0.89941500	-0.00345500
H	2.42144700	0.53932300	-0.88738400

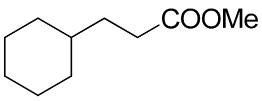

C	-3.53151500	-1.12155500	0.13474200
C	-2.03846200	-1.29781200	0.45140600
C	-1.15418000	-0.28219200	-0.29951800
C	-1.63894000	1.15283500	-0.01425200
C	-3.13220800	1.33555100	-0.33150700
C	-4.00089500	0.31320300	0.41519000
H	-1.88025700	-1.17446700	1.53406100
H	-1.71885000	-2.31962300	0.20639700
H	-3.70591700	-1.35880400	-0.92528500
H	-4.12603500	-1.83858600	0.71529600
H	-1.46255700	1.38077000	1.04876300
H	-1.05120200	1.87973100	-0.58884900
H	-3.44557200	2.35733900	-0.08164400
H	-3.28743300	1.21918300	-1.41440800
H	-3.94108800	0.50863800	1.49628900
H	-5.05529500	0.43180800	0.13452900
H	-1.27741500	-0.46976700	-1.37929400
C	0.32991800	-0.49786800	0.04863500

H	0.49628700	-0.26905400	1.10949200
H	0.57156100	-1.56127200	-0.08449200
C	1.30961900	0.33440400	-0.80599500
H	1.15986900	1.40331100	-0.62194600
H	1.14742700	0.13722300	-1.86989600
C	2.75055400	-0.01397500	-0.49943200
C	4.47970500	0.20104600	1.09035200
H	4.61195900	-0.87994300	1.18835600
H	5.19815900	0.57889400	0.35780200
H	4.62020400	0.69324700	2.05338600
O	3.13683000	0.51304400	0.68867900
O	3.47624100	-0.69559900	-1.19071500

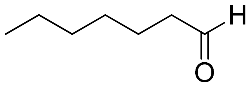

C	3.35450300	-0.24206500	0.29064900
H	3.37140800	-0.87079000	1.21242100
C	0.83222100	-0.29794800	0.10824200
H	0.87885300	-0.98942000	-0.74406800
H	0.81590000	-0.92383700	1.01228400
C	-0.46281500	0.52135800	0.03317100
H	-0.50451500	1.21079900	0.88932500
H	-0.44017900	1.15350700	-0.86618500
C	2.09848600	0.58041600	0.11660000
H	2.18919100	1.16376000	-0.80576000
H	2.03957200	1.28455900	0.96148400
O	4.28668400	-0.26109000	-0.48221000
C	-1.72824500	-0.34544000	0.01431200
H	-1.68409200	-1.03502000	-0.84167100
H	-1.74918200	-0.97891700	0.91363200
C	-3.02591300	0.46984600	-0.06041700
H	-3.00439300	1.10197800	-0.95947100
H	-3.06994500	1.15935600	0.79463700
C	-4.28539400	-0.40224500	-0.07889100
H	-5.19374200	0.20824300	-0.13503900
H	-4.28554900	-1.07988900	-0.94150700
H	-4.35386500	-1.01912400	0.82560800

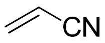

C	-0.57939300	-0.53410000	0.00000000
C	1.32187000	0.98507600	0.00000000
H	2.03285400	0.16489100	0.00000000
H	1.72166300	1.99405700	0.00000000
C	0.00000000	0.77452900	0.00000000
H	-0.70294500	1.60423200	0.00000000
N	-1.07234800	-1.58803000	0.00000000

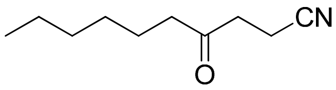

C	1.48005000	0.33108800	0.00021400
C	-1.08064000	0.30797800	0.00020700

H	-1.08519300	0.97048300	0.87465100
H	-1.08531300	0.97034500	-0.87434400
C	-2.33959700	-0.56798500	0.00031400
H	-2.32599000	-1.23062300	-0.87811100
H	-2.32607800	-1.23029300	0.87898700
C	0.21213300	-0.51077100	0.00008900
H	0.25109900	-1.18161000	0.87226900
H	0.25113900	-1.18113600	-0.87247000
O	1.45846800	1.54777600	0.00004200
C	-3.64078600	0.24463700	0.00011700
H	-3.65538900	0.90661800	0.87856600
H	-3.65497200	0.90687500	-0.87814500
C	-4.90520200	-0.62412200	-0.00030800
H	-4.89024300	-1.28560000	0.87767100
H	-4.88993400	-1.28518400	-0.87859500
C	-6.19979800	0.19519700	-0.00031700
H	-7.08360100	-0.45282300	-0.00077500
H	-6.26026100	0.84066800	0.88456600
H	-6.25983900	0.84134100	-0.88473800
C	2.80381900	-0.43262300	0.00052200
H	2.83350400	-1.09566200	0.87523600
H	2.83332100	-1.09684600	-0.87329000
C	4.01294500	0.51738000	-0.00021500
H	3.97322100	1.17468100	-0.87545400
H	3.97374100	1.17538300	0.87451700
C	5.28361400	-0.21125800	-0.00030900
N	6.28197400	-0.80370800	-0.00039600

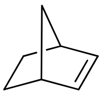

C	-1.27886600	-0.67062700	-0.50778600
C	-0.08736200	-1.12867400	0.32265300
C	-0.08831700	1.12841700	0.32315600
C	-1.27956200	0.66990400	-0.50726200
H	-1.92034800	-1.32910300	-1.08579700
H	-1.92158800	1.32827000	-1.08481800
C	-0.04101100	-0.00042200	1.38045600
H	0.87852400	-0.00012200	1.97942500
H	-0.91211500	-0.00090400	2.04317100
C	1.19173400	-0.78019600	-0.51634400
H	2.08933600	-1.17666500	-0.02775100
H	1.14750200	-1.20639200	-1.52301100
C	1.19079200	0.78143200	-0.51636200
H	2.08804000	1.17899300	-0.02806500
H	1.14574800	1.20746000	-1.52305400
H	-0.11882100	-2.15796400	0.68882600
H	-0.12072700	2.15742800	0.69000900

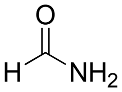

C	0.16205200	0.38759300	0.00000000

H	0.12931500	1.49581800	0.00000200
O	1.20016600	-0.24551600	0.00002000
N	-1.08601800	-0.15816100	-0.00011600
H	-1.91675900	0.41431400	0.00042200
H	-1.18406600	-1.16443500	0.00022900

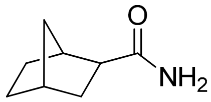

C	1.69229800	1.39277100	0.05800200
C	0.40217100	0.77905500	0.65197000
C	1.54872300	-1.03471600	-0.06614400
C	2.48486000	0.13919300	-0.44038900
H	1.48062100	2.10983100	-0.74369500
H	2.24929200	1.93032000	0.83395000
H	2.69521200	0.17208900	-1.51543900
H	3.44783900	0.05241800	0.07566400
C	0.93089300	-0.53816800	1.25837200
H	0.13453300	-1.18718400	1.63354800
H	1.67130500	-0.37327300	2.05041600
C	-0.47045800	0.25722400	-0.53768700
H	-0.52110300	1.01828800	-1.32627000
C	0.31696800	-1.00940100	-0.99979800
H	-0.30408700	-1.89598600	-0.84341800
H	0.59232300	-0.96075100	-2.05904000
H	-0.13599300	1.44796300	1.33103000
H	2.04888300	-2.00699300	-0.03100400
C	-1.88935200	-0.06981300	-0.07270800
O	-2.21125000	-1.14595200	0.41573800
N	-2.79147800	0.95109700	-0.21616700
H	-3.70906800	0.83340700	0.19042900
H	-2.51602800	1.87293900	-0.51861000

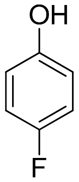

C	-0.70251100	-1.21407100	0.00000000
C	0.69344300	-1.19902200	-0.00000100
C	1.38373000	0.01777700	0.00000000
C	0.67310100	1.22333500	0.00000200
C	-0.71994800	1.21183500	-0.00000300
C	-1.38945100	-0.00737600	0.00000000
H	-1.25540900	-2.14765000	0.00000200
H	1.24294500	-2.13834300	-0.00000300
H	1.22335200	2.15856200	0.00000600
H	-1.28957800	2.13541900	0.00000400
O	2.75120300	0.09506200	-0.00000400
H	3.11818300	-0.80246500	0.00002700
F	-2.74214500	-0.01787500	0.00000000

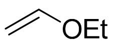

C	2.06119000	0.57956500	-0.00002400
H	1.61327900	1.56629800	0.00013700
H	3.14373100	0.52519800	-0.00007100
C	1.35634300	-0.55668000	-0.00003800
H	1.83858700	-1.53092700	-0.00004900
O	0.00872900	-0.71699300	0.00006600
C	-0.78920200	0.46705500	0.00003800
H	-0.54809100	1.06935500	0.88770600
H	-0.54816500	1.06936400	-0.88766000
C	-2.24807900	0.04562100	-0.00002500
H	-2.47707600	-0.55310100	-0.88731400
H	-2.47758900	-0.55248600	0.88753600
H	-2.89601800	0.92888000	-0.00051800

MeOH

C	0.66232500	-0.01954500	0.00000000
H	1.07971800	0.99103200	-0.00000100
H	1.03695800	-0.54365800	-0.89314700
H	1.03695800	-0.54365600	0.89314900
O	-0.74916800	0.12249700	0.00000000
H	-1.13423600	-0.76642000	0.00000000

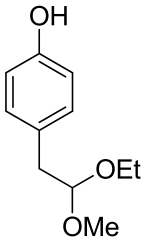

C	-1.22737200	0.43772100	0.89333000
C	-2.51386600	0.94898000	0.72961100
C	-3.45142800	0.25161200	-0.03929800
C	-3.09099700	-0.95728800	-0.64046300
C	-1.80197500	-1.45518000	-0.46525800
C	-0.84657200	-0.77359200	0.30170700
H	-0.50336700	0.99197700	1.48270100
H	-2.79037500	1.89076300	1.20165500
H	-3.82731200	-1.49209800	-1.23226800
H	-1.53473300	-2.40059700	-0.93281000
O	-4.73075400	0.70047700	-0.23428600
H	-4.84254400	1.53972400	0.23848000
C	0.55357800	-1.32478500	0.46732200
H	0.87568800	-1.23671100	1.51155700
C	1.59585800	-0.62426800	-0.41983400
H	1.22373300	-0.55347600	-1.45158700
C	3.51128400	-1.60586200	0.65171100
C	2.49339500	1.55657500	-0.76677300
H	0.56913500	-2.38925900	0.20762200
H	3.47191200	1.13654600	-1.03355600
H	1.91658500	1.67240600	-1.69804900
H	3.02086100	-2.37848800	1.25998400
H	4.49933800	-1.96912400	0.35767800
O	2.80461300	-1.34957000	-0.55595800
O	1.80344700	0.66397500	0.11008400

H	3.62409400	-0.69637100	1.25361400
C	2.64753300	2.89195000	-0.05906100
H	3.22552700	2.77569400	0.86377100
H	3.16828100	3.60732800	-0.70518900
H	1.66810000	3.30745900	0.19970300

HF

F	0.00000000	0.00000000	0.09338400
H	0.00000000	0.00000000	-0.84045800

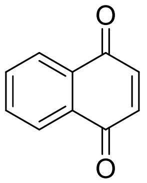

C	-2.67766300	0.69945100	0.00006100
C	-1.47290700	1.40008600	0.00004100
C	-0.26007600	0.70439100	0.00000100
C	-0.25986500	-0.70451300	-0.00012000
C	-1.47262400	-1.40030300	-0.00007500
C	-2.67746900	-0.69985700	0.00001400
H	-3.61899300	1.24189400	0.00013000
C	1.02453700	1.46301700	0.00016800
C	1.02482300	-1.46279800	-0.00039900
H	-1.44733800	-2.48527700	-0.00016800
H	-3.61866600	-1.24256100	0.00002000
C	2.28154100	-0.67159300	-0.00001900
C	2.28139200	0.67192200	0.00005700
H	3.19920000	-1.25358600	0.00006800
H	3.19889200	1.25419300	0.00015200
O	1.06125400	2.68809500	-0.00010300
O	1.06180500	-2.68790900	0.00026600
H	-1.44771100	2.48503700	0.00011900

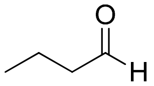

C	-1.46458800	0.22310600	0.28531900
H	-1.51892700	1.10766300	0.96340800
C	1.01488100	0.50453000	-0.14014000
H	0.82737200	0.88186800	-1.15375900
H	0.99472200	1.37990200	0.52384200
C	2.39725500	-0.15223200	-0.08134600
H	2.62438900	-0.50749900	0.93108500
H	2.45577100	-1.01353000	-0.75709900
H	3.18183600	0.55496700	-0.37219800
C	-0.11784000	-0.46245700	0.25585100
H	-0.17590100	-1.31490000	-0.42937800
H	0.08227000	-0.85098200	1.26697900
O	-2.43122300	-0.11439600	-0.36137300

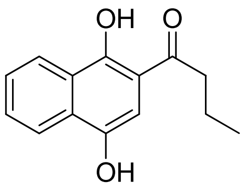

C	0.69831800	-0.36554600	-0.28295100
C	-0.42278700	-1.18999700	-0.07775800
C	-1.73244100	-0.61521100	0.04423000
C	-1.89465100	0.79872700	-0.04073400
C	-0.73591900	1.61779000	-0.24872300
C	0.50406300	1.05311400	-0.36456900
H	-2.73195200	-2.50545500	0.31119100
C	-2.87049600	-1.43194700	0.24897500
C	-3.19106100	1.35135100	0.08160200
H	1.36520100	1.69675700	-0.52041900
C	-4.28409700	0.53127600	0.28136100
C	-4.12466800	-0.86861700	0.36577300
H	-5.27536100	0.96691300	0.37428100
H	-4.99245400	-1.50309500	0.52291000
O	-0.33261400	-2.52122100	0.01311200
H	0.64027000	-2.73678400	-0.09126500
C	2.02186800	-0.98281700	-0.39868900
O	2.16624600	-2.21944700	-0.32003000
C	3.27186800	-0.13648200	-0.57146500
H	4.02167900	-0.78112800	-1.04101600
H	3.09103400	0.70919300	-1.24528200
C	3.81165500	0.37479300	0.78236100
H	3.05062700	0.99515400	1.27284900
H	3.97790800	-0.48849500	1.43841600
C	5.11124900	1.17028000	0.62853800
H	5.89783600	0.55902500	0.17058300
H	4.96581400	2.05342400	-0.00586800
H	5.47907400	1.51507300	1.60131300
O	-0.96368100	2.96960000	-0.32081100
H	-0.11850100	3.42058600	-0.46936000
H	-3.30817900	2.42708800	0.01580400

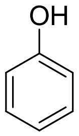

C	1.13169600	1.21940500	-0.00000100
C	-0.26387400	1.19983500	0.00000200
C	-0.94080000	-0.02456700	0.00000100
C	-0.22123400	-1.22431500	0.00000400
C	1.17160400	-1.19037000	-0.00000500
C	1.85730900	0.02761500	0.00000000
H	1.65023200	2.17463600	0.00000200
H	-0.82671600	2.13191200	-0.00000400
H	-0.76605800	-2.16310900	0.00000600
H	1.72555000	-2.12557200	0.00000300
H	2.94309100	0.04676100	0.00000500
O	-2.30694900	-0.11178600	-0.00000400
H	-2.67871500	0.78403800	0.00001400

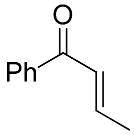

C	0.72659800	0.66271900	-0.04478500
O	0.95581100	1.86497700	-0.15332900
C	3.11641600	0.07745500	-0.02488400
H	3.29554400	1.13786600	-0.20010500
C	1.83880000	-0.31909700	0.07150100
H	1.60986700	-1.36485200	0.25499400
C	4.31224200	-0.81277000	0.08952000
H	4.96461300	-0.48308600	0.90959300
H	4.03490000	-1.85668400	0.26767700
H	4.92020400	-0.76678200	-0.82426200
C	-0.69396000	0.17354200	-0.01502200
C	-1.05497600	-1.17992200	-0.10319700
C	-1.70881600	1.13929200	0.08954100
C	-2.39806700	-1.55704100	-0.08291400
H	-0.29735600	-1.94945600	-0.20691400
C	-3.04749100	0.76305400	0.11796000
H	-1.41547000	2.18221400	0.14705700
C	-3.39592600	-0.58841700	0.03215300
H	-2.66425600	-2.60788600	-0.15829700
H	-3.82192700	1.52041800	0.20518600
H	-4.44151600	-0.88445100	0.05246600

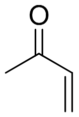

C	-0.44115300	0.16357500	-0.00002500
O	-0.40155900	1.38331300	-0.00000800
C	2.01511900	-0.10445200	0.00000900
H	2.11033900	0.97815700	0.00008200
H	2.92678200	-0.69474800	0.00005500
C	0.80325300	-0.66736700	-0.00002700
H	0.68629500	-1.74976000	-0.00003000
C	-1.75629100	-0.59463000	0.00000800
H	-2.59111300	0.10839900	-0.00025600
H	-1.82259000	-1.24593600	-0.88122600
H	-1.82281400	-1.24537000	0.88164800

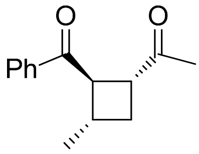

C	1.05843200	1.66106200	-0.84788700
C	0.81882900	0.30016500	-0.08977000
C	2.27952300	-0.10762800	-0.46814300
C	2.58069200	1.38116800	-0.75189500
H	0.73891000	0.48714100	0.98659200
H	2.24595000	-0.71954300	-1.37372400

H	3.19211100	1.60912000	-1.63038300
H	3.02602300	1.84976300	0.13156800
C	3.05365900	-0.80498400	0.63624100
O	3.59633100	-0.17466700	1.52705400
C	3.07696600	-2.32105700	0.59160000
H	2.08039900	-2.72034700	0.36949400
H	3.73303600	-2.64894400	-0.22609600
H	3.45280700	-2.72357300	1.53466000
H	0.72322900	1.54597700	-1.88627400
C	0.50686400	2.95126200	-0.25995300
H	0.82125800	3.07549200	0.78375700
H	0.87747500	3.81722600	-0.82224700
H	-0.58862500	2.97850100	-0.29378800
C	-0.29751400	-0.59751200	-0.59092400
O	-0.05638600	-1.48071500	-1.40594000
C	-1.70284800	-0.36899600	-0.12072000
C	-2.03733000	0.55063300	0.88559000
C	-2.72526800	-1.12362200	-0.72052700
C	-3.36555500	0.71350600	1.28023900
H	-1.26688900	1.13991000	1.37108600
C	-4.04932400	-0.95903200	-0.32837400
H	-2.45130000	-1.83323800	-1.49420400
C	-4.37256500	-0.03888500	0.67401600
H	-3.61254400	1.42607300	2.06231300
H	-4.83198500	-1.54624100	-0.80098100
H	-5.40678200	0.08926200	0.98234900

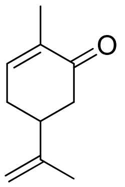

C	-0.88848800	-0.10135800	0.36212500
C	-0.09061400	1.05725800	-0.26859700
C	1.40967000	0.92533900	-0.03160900
C	1.97794900	-0.45090100	-0.00193900
C	1.15784000	-1.51566100	-0.09681400
C	-0.34262000	-1.43892900	-0.18175200
H	-0.24532400	1.07105200	-1.35842300
H	-0.41183700	2.03129400	0.11322200
H	-0.67783300	-0.09163800	1.44016800
H	1.59492200	-2.51463800	-0.11237700
H	-0.64919700	-1.57920900	-1.23078700
H	-0.78881200	-2.27612100	0.37026600
O	2.12518000	1.91011500	0.08809200
C	3.47443300	-0.55774700	0.11897300
H	3.97196900	-0.03099500	-0.70338300
H	3.82386800	-0.08149300	1.04204000
H	3.79972400	-1.60259900	0.11731300
C	-2.39256800	0.04095700	0.19364900
C	-3.19437600	0.06956600	1.26386800
H	-4.27324400	0.16627700	1.16869200
H	-2.80264500	0.00035500	2.27597200
C	-2.95224000	0.15143200	-1.20599700
H	-2.57549500	1.04356800	-1.72288600

H	-2.67719800	-0.71018600	-1.82842600
H	-4.04425800	0.21368000	-1.18756700

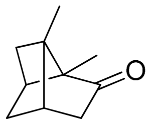

C	-0.52191700	-1.48818300	-1.19285500
C	-0.51680600	-1.39632000	0.36735200
C	0.28261200	0.66192700	-0.30022300
C	-0.57283600	0.02118800	-1.48596900
H	0.36853100	-1.99261900	-1.58505200
H	-0.40901300	0.35299700	-2.51439000
C	-0.94029400	0.11101300	0.53916900
C	0.95239500	-1.42219100	0.81113600
H	1.06360000	-1.31685100	1.89951600
H	1.51060000	-2.31368700	0.50770800
C	1.49391900	-0.15510500	0.13756000
H	-1.16233400	-2.11663700	0.87767000
H	-1.40253300	-2.00157400	-1.59359800
C	-1.83912400	0.41516600	-0.68212000
C	0.56482700	2.15052600	-0.40599600
H	-2.09928400	1.47452900	-0.75470700
H	-2.74022700	-0.19281900	-0.83086200
H	1.43360900	2.30987300	-1.05339400
H	0.81065000	2.57736300	0.57349800
O	2.65946500	0.15910800	0.01195300
H	-0.27887600	2.71175400	-0.81942300
C	-1.27797100	0.60619400	1.93041300
H	-0.48411200	0.38374300	2.65406100
H	-1.43595300	1.69094400	1.93651700
H	-2.19920600	0.13483700	2.29602500

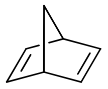

C	1.24590500	0.66762200	-0.52068600
C	0.00000000	1.12267900	0.27150400
C	0.00000000	-1.12267900	0.27150400
C	1.24530800	-0.66827700	-0.52052200
H	1.93709900	1.33834200	-1.01901800
H	1.93619200	-1.33915600	-1.01926100
C	0.00000000	0.00000000	1.35500000
H	-0.89992100	0.00011800	1.97913400
H	0.89992100	-0.00011800	1.97913400
C	-1.24530800	0.66827700	-0.52052200
H	-1.93619200	1.33915600	-1.01926100
C	-1.24590500	-0.66762200	-0.52068600
H	-1.93709900	-1.33834200	-1.01901800
H	-0.00001500	2.16005700	0.61237100
H	0.00001500	-2.16005700	0.61237100

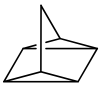

C	0.75759000	0.77511000	-0.71060500
C	0.00000000	1.15217600	0.55114600
C	0.00000000	-1.15217600	0.55114600
C	0.75759700	-0.77511200	-0.71060800
H	1.44997800	1.42738100	-1.23098300
H	1.44996400	-1.42738400	-1.23101100
C	0.00000000	0.00000000	1.54099400
H	-0.89128000	-0.00000100	2.18154400
H	0.89128000	0.00000100	2.18154400
C	-0.75759700	0.77511200	-0.71060800
H	-1.44996400	1.42738400	-1.23101100
C	-0.75759000	-0.77511000	-0.71060500
H	-1.44997800	-1.42738100	-1.23098300
H	0.00000100	2.18810400	0.87787000
H	-0.00000100	-2.18810400	0.87787000

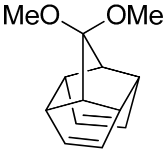

C	-0.90325100	-1.41396700	0.22646400
C	0.06373000	-0.77795800	-0.81444500
C	0.06374600	0.77795900	0.81441200
C	-0.90349300	-0.29512000	1.39838500
H	-0.57272000	-2.37813300	0.62273500
H	-0.56438200	-0.73160800	2.34061700
C	1.09188500	0.00000100	-0.00002700
C	-0.90355700	0.29511500	-1.39840000
H	-0.56449100	0.73159000	-2.34065300
C	-0.90325300	1.41397900	-0.22649500
H	-0.57275100	2.37814500	-0.62280400
H	0.45624300	-1.47144000	-1.56244600
H	0.45628300	1.47144800	1.56239100
O	1.88844000	-0.76538500	0.88410600
O	1.88855800	0.76527400	-0.88409600
C	2.71482200	-1.74253800	0.26202100
C	2.71482400	1.74259600	-0.26202000
H	3.30975400	-1.30769100	-0.54942600
H	3.38271900	-2.12242500	1.03948900
H	2.12495700	-2.57905400	-0.13757600
H	3.38349000	2.12171100	-1.03920000
H	2.12489500	2.57956600	0.13649200
H	3.30893600	1.30811200	0.55021400
C	-2.19361700	-1.46777600	-0.57076500
C	-2.19427000	-0.49699000	-1.49093300
C	-2.19361500	1.46775300	0.57073100
C	-2.19416600	0.49703500	1.49103900
H	-3.01109600	-2.15020800	-0.35657800
H	-3.01281700	-0.24834200	-2.16030100
H	-3.01109500	2.15020300	0.35659400
H	-3.01261200	0.24847800	2.16056100

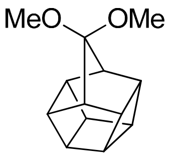

C	0.82267200	1.42917400	-0.21661500
C	-0.07026400	0.47913000	-1.04758600
C	-0.07026300	-0.47919600	1.04757200
C	0.82194400	0.77332500	1.21925700
H	0.56532000	2.48934300	-0.27207000
H	0.55677700	1.42185000	2.05585600
C	-1.10973500	-0.00003800	-0.00001700
C	0.82197800	-0.77336000	-1.21927700
H	0.55686600	-1.42188000	-2.05589600
C	0.82270500	-1.42923200	0.21662100
H	0.56539900	-2.48941000	0.27207900
H	-0.48662000	0.88690200	-1.97197100
H	-0.48666800	-0.88697200	1.97193500
O	-1.90741000	1.00822000	0.58607300
O	-1.90743200	-1.00825300	-0.58606500
C	-2.74915800	1.71886200	-0.31376000
C	-2.74935700	-1.71873400	0.31375700
H	-3.35346800	1.03764600	-0.92385800
H	-3.40746100	2.33678500	0.30236700
H	-2.16979700	2.37340100	-0.97953300
H	-3.40754400	-2.33673200	-0.30241400
H	-2.17009400	-2.37319300	0.97969700
H	-3.35372200	-1.03739800	0.92364000
C	2.32046000	1.03371800	-0.39041500
C	2.31984900	-0.38275600	-1.03619100
C	2.32046100	-1.03367200	0.39038600
C	2.31981600	0.38276000	1.03627300
H	3.04651600	1.77928000	-0.72258000
H	3.04501800	-0.62015000	-1.81799200
H	3.04658600	-1.77917700	0.72254600
H	3.04497500	0.62009800	1.81809600

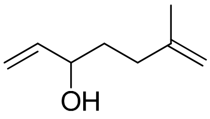

C	-3.55250600	0.48945900	-0.72240700
C	-2.60013300	-0.44324200	-0.72590800
H	-4.33859100	0.50558100	-1.47174600
H	-3.56629000	1.27665500	0.02646300
H	-2.59468000	-1.21598900	-1.49557200
C	-1.47305600	-0.52951200	0.27813300
H	-1.40976400	-1.57595000	0.63078500
C	-0.12378400	-0.16499600	-0.35794100
H	0.01450000	-0.76775700	-1.26465900
H	-0.18276600	0.88300200	-0.67440500
C	1.06868500	-0.38444400	0.59385400
H	0.89815400	0.21538700	1.49706000
H	1.08250700	-1.43623700	0.90808800
C	2.40696500	-0.02580900	-0.01783500
C	3.31594500	-0.96240200	-0.30956500

H	4.27828300	-0.71075600	-0.74919700
H	3.13283200	-2.01692800	-0.11613300
C	2.66786800	1.43956000	-0.27388800
H	2.57594100	2.02084900	0.65388200
H	3.66934500	1.60485300	-0.68294600
H	1.94192600	1.86452800	-0.97937900
O	-1.65788000	0.34256500	1.39007400
H	-2.49826000	0.10056000	1.81050400

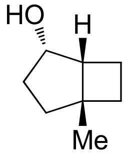

C	1.35635300	-0.35600300	-0.51657800
C	0.14554600	0.58445900	-0.70147700
C	-1.08665200	-0.15653900	-0.05268000
C	-0.52505600	-1.49369000	0.48187000
H	0.02579900	0.90482600	-1.74333200
C	-1.16247300	0.97544000	1.02497600
C	0.02134800	1.71997500	0.34994900
H	-0.97025400	0.63843300	2.04998500
H	-0.24664800	2.69194700	-0.07585800
H	0.91467000	1.85226700	0.96712200
H	-2.10955000	1.52577400	1.02370200
H	-1.02879500	-1.81871600	1.40043000
H	-0.68019600	-2.28383100	-0.26636600
H	1.17501300	-0.69258900	1.60053400
H	1.57532500	-2.16591400	0.71452700
H	1.44687500	-0.98765900	-1.41689200
C	0.98030900	-1.24795400	0.67417800
O	2.59779900	0.29104900	-0.25425100
H	2.81644000	0.83435700	-1.02736300
C	-2.34314100	-0.31691800	-0.89967400
H	-3.16341100	-0.74645400	-0.30917000
H	-2.68905300	0.64773200	-1.29127900
H	-2.16601000	-0.98117600	-1.75542100

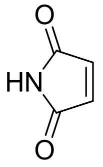

C	-0.00028500	-0.15706000	1.15773500
C	0.00100900	1.26545400	0.66861300
C	0.00100900	1.26545400	-0.66861300
C	-0.00028500	-0.15706000	-1.15773500
H	0.00208300	2.10219900	-1.35548900
H	0.00208300	2.10219900	1.35548900
O	-0.00028500	-0.56083200	2.30013900
O	-0.00028500	-0.56083200	-2.30013900
N	-0.00085500	-0.94015400	0.00000000
H	-0.00231600	-1.95074700	0.00000000

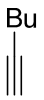

C	-2.15358900	0.05954100	-0.00293000
C	-0.85265000	0.73008100	-0.00021900
H	-0.78148400	1.39078100	-0.87607200
H	-0.78359400	1.38593300	0.87942600
C	-3.22116200	-0.50526400	0.00188300
H	-4.16287800	-1.00495300	0.00451100
C	0.33917200	-0.25056100	-0.00162200
H	0.26266000	-0.90635800	0.87536700
H	0.26444200	-0.90179900	-0.88215900
C	1.69305000	0.46850000	0.00158200
H	1.75398300	1.12420800	0.88191300
H	1.75568400	1.12890300	-0.87511000
C	2.88234800	-0.49724100	0.00012200
H	3.83540200	0.04356600	0.00246500
H	2.86554000	-1.14766100	0.88311400
H	2.86722300	-1.14295700	-0.88635000

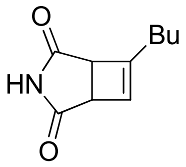

C	-1.36840300	1.37207300	0.09804300
C	-0.71149000	0.17028200	0.76573200
C	-1.78078100	-0.96474800	0.73029800
C	-3.00482300	-0.34472300	0.06171800
O	-0.89177100	2.46523300	-0.12017800
O	-4.07715400	-0.85509200	-0.17491400
N	-2.65657600	0.97119800	-0.26345500
H	-3.28260200	1.57899400	-0.78067900
H	-0.25821300	0.43498100	1.72532600
H	-2.04414400	-1.46669200	1.66586600
C	-0.81926200	-1.68451200	-0.21210800
C	0.10857200	-0.71313900	-0.18970000
H	-0.90468700	-2.63392700	-0.73221700
C	1.43154500	-0.46724100	-0.83358000
H	1.65816000	-1.29419000	-1.51934400
H	1.36301800	0.44468200	-1.44552400
C	2.58241100	-0.29307000	0.17828300
H	2.34323600	0.53712600	0.85708300
H	2.65547600	-1.19456400	0.80235400
C	3.93246700	-0.02267400	-0.49716500
H	4.16946500	-0.85188700	-1.17867300
H	3.84787600	0.87490800	-1.12562200
C	5.07682300	0.15955400	0.50501400
H	4.88394100	1.00475000	1.17684700
H	5.20646300	-0.73507500	1.12633300
H	6.02708100	0.35055800	-0.00604300

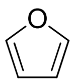

C	1.09501500	-0.34743500	0.00051100
C	0.71791100	0.95993700	-0.00029100
C	-0.71802100	0.95986000	0.00005700
C	-1.09497300	-0.34755600	0.00027600
O	0.00006500	-1.16096700	-0.00052300
H	2.05026900	-0.84991500	0.00084200
H	1.37359800	1.81954700	-0.00052300
H	-1.37380400	1.81939700	0.00009600
H	-2.05017600	-0.85013300	0.00044800

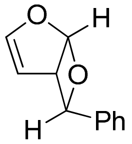

C	1.87793300	-0.91078600	0.33859700
C	1.33787500	0.50710900	0.64199700
C	2.54755300	1.35630800	0.35907700
C	3.55335300	0.54961600	0.00815400
O	3.27011200	-0.79697500	0.04033600
H	0.83457800	0.69745200	1.59374900
H	2.58998500	2.43584000	0.41447100
H	4.57223900	0.78259800	-0.27596400
H	1.76423500	-1.75262500	1.02583700
C	0.39307500	0.23585900	-0.57601200
H	0.56958500	0.91755300	-1.41674000
C	-1.08371300	0.11318300	-0.29718900
C	-1.67085100	-1.11858800	0.00935700
C	-1.88465400	1.26330900	-0.31702100
C	-3.03458300	-1.19760800	0.29840800
H	-1.05551600	-2.01236900	-0.00267300
C	-3.24459000	1.18600900	-0.02029000
H	-1.44077300	2.22464200	-0.56921800
C	-3.82407400	-0.04704000	0.28929800
H	-3.48086100	-2.16189600	0.52758900
H	-3.85385400	2.08590600	-0.03908100
H	-4.88534700	-0.10991800	0.51450300
O	1.05261100	-1.04445100	-0.81267700

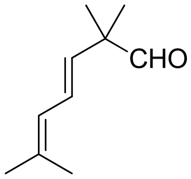

C	2.59009300	-0.38318300	-1.04801600
O	3.49957800	-1.18074800	-1.08041200
H	2.08459700	-0.05064000	-1.98384100
C	1.99042700	0.23352300	0.22556800
C	0.62627800	-0.42088300	0.35051100
C	-0.57253200	0.13170600	0.08225400
H	0.65792900	-1.46575700	0.66378900
H	-0.61785300	1.16998600	-0.23612900
C	-1.82771200	-0.59065200	0.21059300
C	-3.07461200	-0.12587500	-0.02210100

H	-1.73133800	-1.62844700	0.53174600
C	2.86285000	-0.11733700	1.44179600
C	1.91248800	1.75679700	0.01758600
H	3.87829000	0.27255600	1.31577500
H	2.43344400	0.31041800	2.35397800
H	2.94073700	-1.20118700	1.56841700
H	1.39715000	2.01746200	-0.91425600
H	1.37256300	2.22809900	0.84587300
H	2.91796000	2.18971200	-0.02534200
C	-3.40120000	1.27584500	-0.47009800
C	-4.27268000	-1.02313600	0.15904500
H	-2.52235300	1.91219300	-0.59468500
H	-3.94129200	1.25875300	-1.42706000
H	-4.06943700	1.76504000	0.25236200
H	-3.98728400	-2.02798600	0.48505200
H	-4.96873200	-0.60807900	0.90201800
H	-4.84140700	-1.11696700	-0.77722800

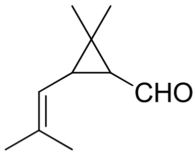

C	-0.05305500	0.02072400	0.25766500
C	-1.32599100	-0.39076500	-0.52306800
C	-1.19391500	0.99842500	0.10551900
H	0.00212700	-0.42547900	1.24828500
H	-1.23116000	-0.43417400	-1.60572900
C	1.23817200	0.12938300	-0.45728900
C	2.45811000	-0.20138400	0.00117900
H	1.17897000	0.51476500	-1.47414100
C	3.67664800	-0.02769200	-0.87332500
C	2.74964400	-0.75775100	1.37284800
C	-1.07806500	2.16423800	-0.86424500
C	-1.99586500	1.34791400	1.35173600
C	-2.18952300	-1.45837700	0.02351400
H	-2.07526800	2.52893500	-1.13876500
H	-0.53174300	2.99640800	-0.40281400
H	-0.55972100	1.89446600	-1.78843900
H	3.42145500	0.37786000	-1.85740000
H	4.40639300	0.64857200	-0.40577600
H	4.19459900	-0.98539800	-1.02440000
H	3.24522400	-1.73532500	1.29504500
H	3.44408400	-0.10138600	1.91566300
H	1.85999200	-0.88498800	1.99392700
H	-2.01413800	0.53653200	2.08540800
H	-1.55621200	2.22504500	1.84266100
H	-3.03404900	1.59454300	1.09670700
H	-2.26736400	-1.49585000	1.13357600
O	-2.77501800	-2.27535200	-0.66012600

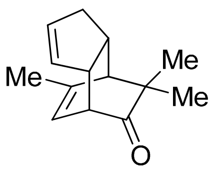

C	-0.31017800	0.61788900	-1.56952200
C	-0.04214300	-0.84326900	-1.28357700
C	0.19109400	0.72108900	0.76928600
C	-0.19413200	1.44133400	-0.51736700
C	1.37476600	-0.90611000	-0.70617700
C	1.56317700	0.00105000	0.53191200
H	0.28296000	1.41962500	1.61066000
H	-0.10667000	-1.48322400	-2.16662500
C	-0.91382400	-0.33687100	1.06950200
H	-0.63773700	-0.88445300	1.97407700
C	-1.00497500	-1.32967800	-0.14051900
H	-0.69145000	-2.34368900	0.14544100
H	-0.56758100	0.95322300	-2.57085900
C	2.01315700	-0.87783500	1.71839800
C	2.68721100	1.01798600	0.24238100
H	2.98106700	-1.33251900	1.48686900
H	2.12363200	-0.26941700	2.62441000
H	1.31551100	-1.69131900	1.93837800
H	3.60424800	0.48789200	-0.03226800
H	2.42764700	1.68943900	-0.58136500
H	2.89169400	1.62469900	1.13321900
O	2.26295300	-1.60203900	-1.15746000
C	-0.40168700	2.92738100	-0.54564900
H	0.50925000	3.46209800	-0.24276700
H	-0.68373800	3.27548100	-1.54494000
H	-1.18865600	3.23556600	0.15656200
C	-2.34695600	0.24886400	1.25941300
C	-3.17845100	-0.46170900	0.22076200
C	-2.46335700	-1.30770300	-0.52301700
H	-2.37812100	1.33831000	1.11913900
H	-2.72463700	0.06598300	2.27516000
H	-4.24573300	-0.28614500	0.11020100
H	-2.85751700	-1.91974000	-1.33057300

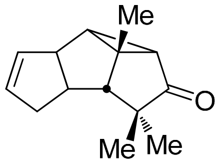

C	-1.56571500	-0.60118300	-0.41663100
C	-1.72910300	0.11258200	0.94145500
C	-0.78153900	1.26648000	0.99328300
C	0.04466600	1.28890000	-0.28538600
C	-0.16282300	-0.12830400	-0.91728400
H	-1.12378200	2.16932900	1.49270300
H	-0.15020700	-0.08783000	-2.01249100
C	-2.65516700	0.00042700	-1.33959600
C	-1.79645900	-2.11065800	-0.28944100
H	-2.53825600	1.08371700	-1.45583000
H	-2.59540800	-0.45477400	-2.33576900

H	-3.65263500	-0.19083600	-0.93054800
H	-2.81602000	-2.29324500	0.06417100
H	-1.67016600	-2.61459400	-1.25526000
H	-1.12002100	-2.57774100	0.43260900
O	-2.56328200	-0.17025300	1.77993700
C	0.73425600	1.01851500	1.01778800
C	1.01846000	-0.96393200	-0.37501000
C	0.25122200	2.52205600	-1.13167800
H	0.38980600	3.40933800	-0.50312300
H	1.14064300	2.42412400	-1.76698100
H	-0.60645400	2.71139900	-1.78903600
C	1.32426800	-0.39908200	1.05244500
H	0.88267400	-0.99956300	1.85894100
H	0.77951100	-2.02929700	-0.33144300
H	1.29711300	1.81329900	1.50324600
C	2.83558400	-0.38332000	1.11259700
C	2.35351400	-0.80192900	-1.16354100
H	2.56443000	-1.67835700	-1.79017800
H	2.32728300	0.06017500	-1.84744100
C	3.38878100	-0.59120900	-0.08448600
H	4.45635100	-0.58360300	-0.28943900
H	3.38172700	-0.17556600	2.02928100

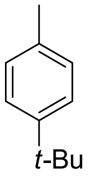

C	0.36303900	1.21791600	-0.00254500
C	1.76082000	1.20458700	-0.00731200
C	2.47770300	0.00623100	-0.00714100
C	1.73925400	-1.18560900	-0.00738100
C	0.34789200	-1.17185000	-0.00297400
C	-0.37988800	0.03144800	0.00025200
H	-0.14130900	2.17819600	-0.00422300
H	2.29962900	2.15002800	-0.01238400
H	2.26272300	-2.13987700	-0.01266100
H	-0.17942900	-2.12230600	-0.00539100
C	3.98839200	-0.01217900	0.01073900
C	-1.91924300	0.00306400	0.00187200
H	4.40339600	0.96603800	-0.25326300
H	4.37457500	-0.27498200	1.00470700
H	4.39014500	-0.74949400	-0.69419600
C	-2.42333600	-0.72876700	-1.26467300
C	-2.53138000	1.41674900	0.01262600
C	-2.42074700	-0.74642800	1.25904600
H	-2.24147100	1.99415500	-0.87268400
H	-3.62491600	1.34474700	0.01629000
H	-2.23435100	1.98328800	0.90260600
H	-2.08682400	-0.24348900	2.17378700
H	-3.51716400	-0.78275000	1.27143300
H	-2.05343400	-1.77746800	1.29256900
H	-2.05555700	-1.75904100	-1.31332300
H	-3.51979300	-0.76514100	-1.27548200
H	-2.09124700	-0.21287400	-2.17284300

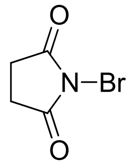

C	0.91538800	-1.19119600	-0.00002000
C	2.38284400	-0.76832600	0.00066700
C	2.38170500	0.77113700	0.00025300
C	0.91322200	1.19145700	-0.00002300
H	2.86605100	-1.20214400	0.88133600
H	2.86464700	1.20564800	-0.88020700
H	2.86700300	-1.20276400	-0.87918000
H	2.86470400	1.20615600	0.88040700
O	0.46211600	-2.30806700	-0.00154200
O	0.45849200	2.30767800	-0.00136300
N	0.16662000	-0.00042900	0.00140700
Br	-1.70150200	-0.00054900	0.00016500

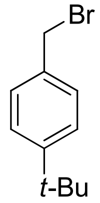

C	-1.14619000	1.25356200	-0.19282100
C	0.21265300	1.28361000	-0.50760000
C	0.91776700	0.10410100	-0.76539700
C	0.21985500	-1.10995800	-0.69513300
C	-1.13397400	-1.13471200	-0.38040000
C	-1.85367700	0.04565300	-0.12028900
H	-1.65139300	2.19412200	-0.00456000
H	0.72995500	2.23940100	-0.55178300
H	0.74477000	-2.04314400	-0.88642500
H	-1.63940100	-2.09543200	-0.33905800
C	2.36445600	0.13760700	-1.12270400
C	-3.35228100	-0.02731100	0.22253100
H	2.67096100	1.08592400	-1.56161200
H	2.66176900	-0.68650900	-1.76973100
C	-3.54599700	-0.87873800	1.50004800
C	-3.96383900	1.36353700	0.47648900
C	-4.11638200	-0.68344300	-0.95229900
H	-3.48466000	1.87237100	1.32059500
H	-5.02799200	1.25954500	0.71586100
H	-3.88590100	2.01153200	-0.40394300
H	-4.00345900	-0.09569200	-1.87041900
H	-5.18629900	-0.75024900	-0.72048700
H	-3.75674500	-1.69708800	-1.15821000
H	-3.17185200	-1.89969700	1.37153900
H	-4.61060500	-0.94356300	1.75593400
H	-3.01813500	-0.43414700	2.35128400
Br	3.57184700	-0.06116600	0.48504200

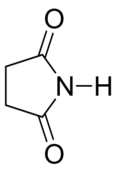

C	0.00000600	-0.21466500	1.17834200
C	0.00000600	1.25975000	0.76974100
C	0.00000600	1.25975000	-0.76974100
C	0.00000600	-0.21466500	-1.17834200
H	-0.87937500	1.74379900	-1.20568900
H	-0.87937500	1.74379900	1.20568900
O	0.00001000	-0.67695500	2.29689700
O	0.00001000	-0.67695500	-2.29689700
N	-0.00003200	-0.95897500	0.00000000
H	0.00012700	-1.97232700	0.00000000
H	0.87927200	1.74390600	1.20576900
H	0.87927200	1.74390600	-1.20576900

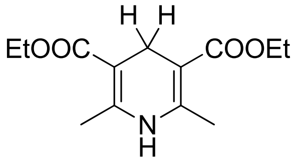

C	-1.23290200	1.28766900	-0.00038300
C	-1.27179000	-0.07108100	-0.00045500
C	-0.00001500	-0.90548700	-0.00075800
C	1.27178100	-0.07111900	-0.00037200
C	1.23293900	1.28762700	-0.00029800
N	0.00002900	1.92662300	-0.00049600
H	-0.00009900	-1.58548100	0.86165800
C	2.40134600	2.24538900	0.00010700
C	-2.40124500	2.24550100	-0.00023300
C	-2.49794000	-0.88182100	-0.00013200
C	2.49792900	-0.88185900	-0.00029100
H	3.03609900	2.09474300	-0.87587500
H	2.05117900	3.28392700	-0.00044500
H	3.03502300	2.09530300	0.87697800
H	-3.03493600	2.09570800	0.87668300
H	-2.05099800	3.28401200	-0.00106900
H	-3.03599600	2.09465700	-0.87617500
H	0.00004600	-1.58485000	-0.86367800
O	2.48362400	-2.10196900	-0.00067200
O	-2.48361000	-2.10192400	-0.00029600
O	-3.66648000	-0.17626900	0.00055400
O	3.66643500	-0.17627100	0.00034100
C	4.86670100	-0.97636600	0.00025000
C	-4.86673400	-0.97635700	0.00092100
H	-4.86263200	-1.62498400	0.88300000
H	-4.86262900	-1.62592300	-0.88045600
H	4.86287400	-1.62500000	-0.88181700
H	4.86232800	-1.62591000	0.88164300
C	6.05080200	-0.02668800	0.00106600
C	-6.05087600	-0.02671600	0.00043400
H	-6.98590500	-0.59765200	0.00075800
H	-6.04084600	0.61486600	0.88792300

H	-6.04085200	0.61383400	-0.88780900
H	6.98584500	-0.59759900	0.00129300
H	6.04113700	0.61462400	-0.88662900
H	6.04038700	0.61412900	0.88910300
H	0.00004400	2.93456200	-0.00016300

H_2

H	0.00000000	0.00000000	0.37139400
H	0.00000000	0.00000000	-0.37139400

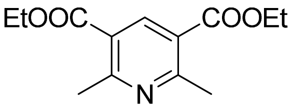

C	1.17314800	1.38206600	0.00017200
C	1.21711900	-0.03241100	0.00009200
C	-0.00000300	-0.71360800	-0.00001600
C	-1.21712200	-0.03240700	-0.00006800
C	-1.17314600	1.38207100	-0.00015300
N	0.00000300	2.03480400	-0.00003000
C	-2.38229300	2.28405900	-0.00051200
C	2.38230500	2.28404200	0.00039000
C	2.43709500	-0.89903100	0.00007800
C	-2.43709800	-0.89902900	-0.00009900
H	-3.01285800	2.10756800	0.87625000
H	-2.03706300	3.31937200	-0.00115800
H	-3.01317500	2.10654000	-0.87683300
H	3.01314900	2.10707500	-0.87606900
H	2.03708600	3.31935900	0.00040000
H	3.01290200	2.10698300	0.87701500
O	-2.39166400	-2.11428000	-0.00047300
O	2.39166200	-2.11428000	0.00004600
O	3.59935300	-0.21233500	0.00009000
O	-3.59935600	-0.21233800	0.00038600
C	-4.80136500	-1.01960600	0.00033300
C	4.80135900	-1.01960400	0.00007700
H	4.78817400	-1.66666200	-0.88246500
H	4.78847300	-1.66622800	0.88295000
H	-4.78845500	-1.66629100	0.88316400
H	-4.78820600	-1.66660400	-0.88225200
C	-5.98730400	-0.07387400	0.00003700
C	5.98730200	-0.07387400	-0.00030400
H	6.91887700	-0.65012200	-0.00031400
H	5.97959000	0.56637900	-0.88831500
H	5.97984600	0.56673600	0.88745700
H	-6.91888400	-0.65011200	0.00000000
H	-5.97982900	0.56669800	0.88782500
H	-5.97958900	0.56642700	-0.88794300
H	-0.00000500	-1.79763800	-0.00007400

CH_4

C	0.00000000	0.00000000	0.00000000
H	0.63133900	0.63133900	0.63133900

H	-0.63133900	-0.63133900	0.63133900
H	-0.63133900	0.63133900	-0.63133900
H	0.63133900	-0.63133900	-0.63133900

CO_2

C	0.00000000	0.00000000	0.00000000
O	0.00000000	0.00000000	1.16915600
O	0.00000000	0.00000000	-1.16915600

References

1. Frisch, M.J.; Trucks, G.W.; Schlegel, H.B.; Scuseria, G.E.; Robb, M.A.; Cheeseman, J.R.; Scalmani, G.; Barone, V.; Mennucci, B.; Petersson, G.A.; *et al. Gaussian 09*, *Version D.01*; Gaussian, Inc.: Wallingford, CT, USA, 2009.
2. Becke, A.D. Density-Functional Thermochemistry. III. The Role of Exact Exchange. *J. Chem. Phys.* **1993**, *98*, 5648–5652.
3. Lee, C.; Yang, W.; Parr, R.G. Development of the Colle-Salvetti Correlation-Energy Formula into a Functional of the Electron Density. *Phys. Rev. B* **1988**, *37*, 785–789.
4. Vosko, S.H.; Wilk, L.; Nusair, M. Accurate Spin-Dependent Electron Liquid Correlation Energies for Local Spin Density Calculations: A Critical Analysis. *Can. J. Phys.* **1980**, *58*, 1200–1211.
5. Stephens, P.J.; Devlin, F.J.; Chabalowski, C.F.; Frisch, M.J. Ab Initio Calculation of Vibrational Absorption and Circular Dichroism Spectra Using Density Functional Force Fields. *J. Phys. Chem.* **1994**, *98*, 11623–11627.
6. Baboul, A.G.; Curtiss, L.A.; Redfern, P.C.; Raghavachari, K. Gaussian-3 theory using density functional geometries and zero-point energies. *J. Chem. Phys.* **1999**, *110*, 7650–7657.
